# Impairment of spinal CSF flow precedes immune cell infiltration in an active EAE model

**DOI:** 10.1186/s12974-024-03247-9

**Published:** 2024-10-23

**Authors:** Li Xin, Adrian Madarasz, Daniela C. Ivan, Florian Weber, Simone Aleandri, Paola Luciani, Giuseppe Locatelli, Steven T. Proulx

**Affiliations:** 1 Theodor Kocher Institute, University of Bern, Freiestrasse 1, Bern, CH-3012 Switzerland; 2 Department of Chemistry, Biochemistry and Pharmaceutical Sciences, University of Bern, Bern, Switzerland

**Keywords:** Cerebrospinal fluid, CSF flow, Neuroinflammation, Subarachnoid space, Experimental autoimmune encephalomyelitis, Glia limitans, Multiple sclerosis

## Abstract

Accumulation of immune cells and proteins in the subarachnoid space (SAS) is found during multiple sclerosis and in the animal model experimental autoimmune encephalomyelitis (EAE). Whether the flow of cerebrospinal fluid (CSF) along the SAS of the spinal cord is impacted is yet unknown. Combining intravital near-infrared (NIR) imaging with histopathological analyses, we observed a significantly impaired bulk flow of CSF tracers within the SAS of the spinal cord prior to EAE onset, which persisted until peak stage and was only partially recovered during chronic disease. The impairment of spinal CSF flow coincided with the appearance of fibrin aggregates in the SAS, however, it preceded immune cell infiltration and breakdown of the glia limitans superficialis. Conversely, cranial CSF efflux to cervical lymph nodes was not altered during the disease course. Our study highlights an early and persistent impairment of spinal CSF flow and suggests it as a sensitive imaging biomarker for pathological changes within the leptomeninges.

**Supplementary Information:**

The online version contains supplementary material available at 10.1186/s12974-024-03247-9.

## Background

Multiple sclerosis (MS) is a demyelinating, neuroinflammatory autoimmune disease of the central nervous system (CNS) [1]. One of the hallmarks of early or relapse-remitting MS (RRMS) is the development of focal white matter lesions that are associated with blood-brain-barrier (BBB) leakage, an accumulation of leukocytes at perivascular inflammatory cuffs and infiltration of these cells into the parenchyma through breakdown of the glia limitans. Numerous studies have also documented immune cell infiltrates in the leptomeninges of MS patients, particularly in the context of compartmentalized disease progression [2–4]. Leptomeningeal inflammation varies from unorganized clusters of myeloid cells, T and B lymphocytes to tertiary lymphoid structures consisting of follicular dendritic cells, follicular helper T cells and B cells. Moreover, neuropathological and imaging studies have suggested a “surface-in” gradient spatial distribution of brain lesions, such that periventricular and subpial cortical regions show lesions that decrease in severity with distance from the CSF compartment [5–8]. Studies from the animal model experimental autoimmune encephalomyelitis (EAE) have also highlighted involvement of the leptomeninges during neuroinflammation [9]. Schlager and colleagues described an entry of leukocytes at the leptomeningeal vessels of the spine and an accumulation in the CSF over time using two-photon imaging [10]. A recent study using tissue from MS patients and EAE models demonstrated more significant involvement in the leptomeninges compared to the dura mater [11].

Notably, changes in the composition of CSF of MS patients (e.g. cytokine, chemokine, IgG and IgM levels) have been used as key disease biomarkers, assisting in the diagnosis of MS and allowing for the stratification of MS patients and improved understanding of pathophysiological mechanisms. For example, upregulation of proinflammatory cytokines in the CSF of MS patients has been suggested to support the role of Th1 lymphocytes in the pathogenesis of neuroinflammation. Neurofilament light chain levels in the serum and CSF has been utilized as a biomarker for both inflammation and neurodegeneration in MS [12–18]. Nevertheless, it is largely unknown whether the flow dynamics of CSF are impacted due to pathological changes in the leptomeninges. Our understanding of how CSF circulates and is cleared from the CNS has greatly evolved in the last few years. CSF is mainly produced in the ventricles of the brain by the choroid plexuses [19]. From the fourth ventricle, a portion of CSF flows down the central canal of the spinal cord, while the majority passes through the foramen of Magendie and Luschka into the SAS. From the SAS it is now considered that the majority of CSF is cleared via the lymphatic system through routes existing to lymphatic vessels in the dura mater and along some cranial nerves to cervical lymph nodes (cLNs) before it reaches the systemic circulation [20–22]. In many animal models of neurological conditions, as well as during the normal aging process, it is becoming apparent that alterations in CSF flow and efflux occur [22–24]. Neuroradiological findings from the clinic support that this concept may also apply to MS. In subjects with MS, phase-contrast MRI studies have revealed significantly decreased CSF flow through the cerebral aqueduct compared to healthy controls [25, 26]. In a more recent study, Onener et al. observed significantly higher peak velocities and caudocranial and craniocaudal CSF flow volumes in MS patients [27]. Nevertheless, other studies have found no difference between MS patients and healthy subjects in net CSF flow volume or flow rate passing through cerebral aqueduct [28, 29]. However, at this point in time, our understanding of the changes occurring in CSF dynamics in MS patients is limited by the lack of longitudinal imaging data and the restrictions on the use of intrathecal-administered contrast agents in clinical studies. In addition, the lack of histopathological assessments at the early stage of MS disease hampers the appreciation of the mechanisms underlying the radiological changes.

Using non-invasive intravital near-infrared (NIR) imaging, our group has previously demonstrated that besides cranial CSF circulation, a minor portion of CSF flows in a rostral-caudal direction towards the sacral spinal cord of mice [30], which was later confirmed by MRI imaging [31]. In the current study, we hypothesized that immune cell infiltrates and protein aggregates in the SAS may perturb the bulk flow of CSF during EAE disease development. Consequently, we sought to assess the flow of intracerebroventricularly (i.c.v) infused CSF tracers along the spinal cord and CSF tracer efflux to cervical LNs and to the systemic circulation by intravital NIR imaging at several EAE disease phases including before and during onset, at the clinical peak of disease and during the resulting chronic phase. We then confirmed the findings from NIR imaging by assessing the distribution of CSF tracers on decalcified sections. We also closely examined the tissue at different stages of disease for CCR2^+^ immune cell infiltrates, the breakdown of glia limitans superficialis, fibrin aggregates in the SAS and drainage function of nasal lymphatics.

## Results

### Impaired cranial-to-caudal CSF flow along the spinal cord during EAE

We first set out to evaluate the dynamics of CSF flow across the disease course in MOG_35 − 55_/CFA-PTX-induced active EAE in double knock-in *CCR2-RFP x CX3CR1-GFP* mice [32]. The expression of distinct reporter proteins in this line allows distinguishing blood-borne inflammatory monocyte-derived cells (CCR2^high^, CX3CR1^low^) from tissue-resident microglia and border-associated macrophages (BAMs) (CCR2^negative^, CX3CR1^high^). Mice were first subjected to NIR imaging to assess the flow and clearance of CSF administered tracers, then were perfused and decalcified for histopathological assessments of the spinal cord. Since the 40 kDa PEGylated P40D800 tracer [33] used for NIR imaging is not amenable to fixation, we co-infused either ovalbumin-AF647(OVA-AF647) [31] or custom-designed fluorescently-labelled liposomes [34] into the lateral ventricle, both of which are efficiently taken up by BAMs and preserved during fixation for histological analysis. OVA-AF647 has a similar molecular size to the P40D800 tracer, and thus serves as a marker to confirm the NIR imaging findings.

**Fig. 1:**
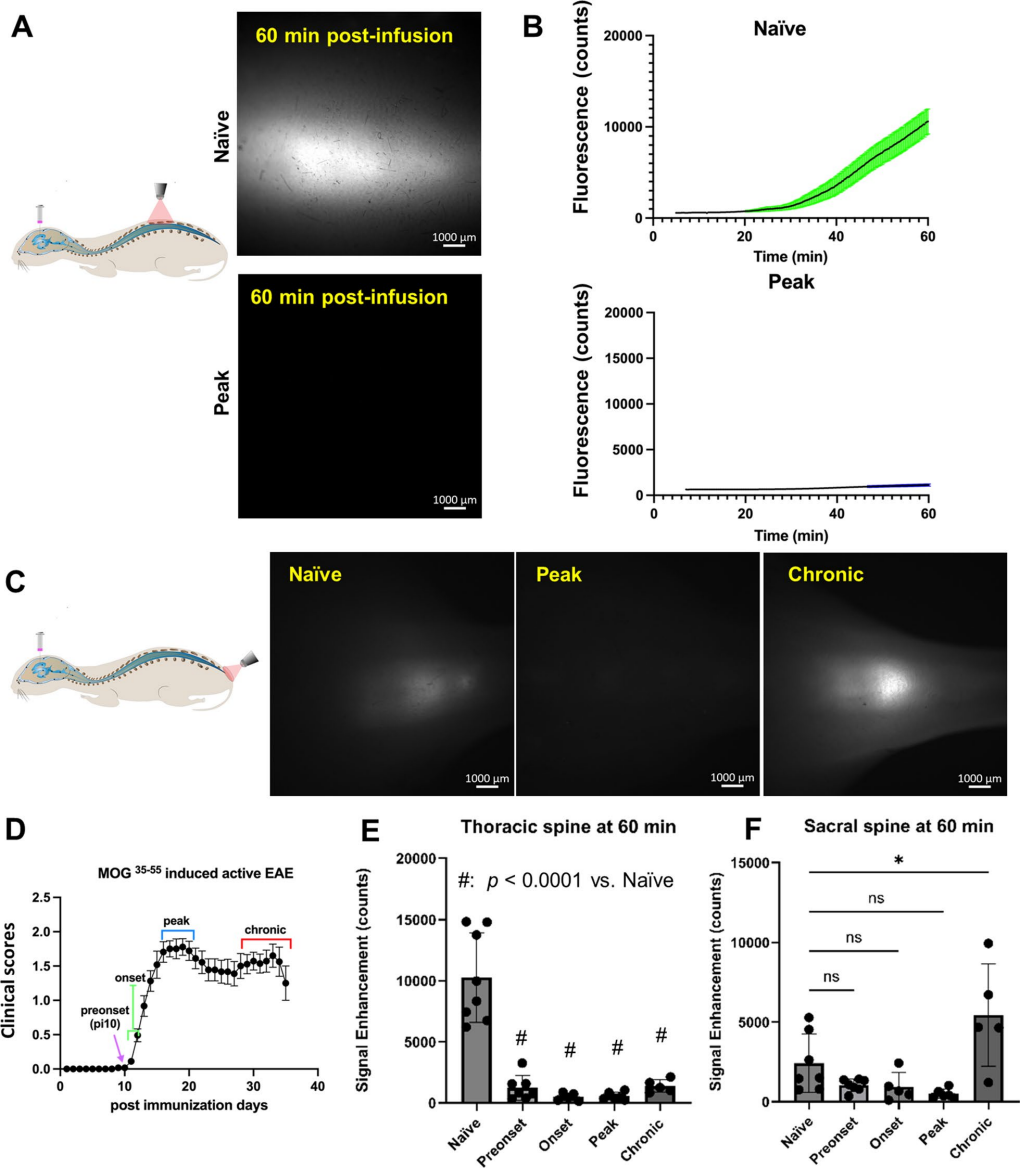
Impaired CSF flow along the spinal cord in EAE mice. **(A)** Representative images of P40D800 tracer within the thoracic spine at 60 min post-i.c.v infusion into naïve mice and mice of EAE peak stage showing impaired CSF flow at peak EAE. **(B)** P40D800 tracer fluorescence intensity counts within the thoracic spine plotted against post-infusion time in naïve and EAE peak groups (*n* = 8 naïve, *n* = 6 peak). **(C)** Representative pictures of P40D800 tracer within the sacral region of the spine 60 min post i.c.v-infusion. **(D)** Average clinical scores of active EAE mice (*n* = 29 mice) illustrating the time-points evaluated during EAE development. **(E)** Quantification of fluorescence signal enhancement in the thoracic region at 60 min post-i.c.v infusion. Data are presented as mean ± SD (*n* = 8 naïve, *n* = 7 preonset, *n* = 5 onset, *n* = 6 peak, *n* = 5 chronic mice). **(F)** Quantification of fluorescence signal enhancement in the sacral region (*n* = 7 naïve, *n* = 7 preonset, *n* = 5 onset, *n* = 6 peak, *n* = 5 chronic mice). Statistics with one-way ANOVA with Dunnett’s post hoc test (ns, no statistical difference; *, *p* < 0.1)

Using NIR imaging after i.c.v-infusion, the P40D800 tracer within the spinal SAS can be detected through the shaved back skin without surgical intervention [30]. The spread of the tracer to the region between vertebrae T10 and T12 (imaging schematic, Fig. 1A), which is equivalent to spinal cord segment T12-L3 [35] was recorded for 60 min after tracer infusion. As shown in Fig. 1D, representing a clinical score curve of our EAE disease model (*n* = 29 mice), we selected 4 stages to evaluate including post-immunization day 10 when mice showed no clinical symptoms (preonset), as well as onset (post-immunization day, p.i. 11 and 12), peak (p.i. 17–21) and chronic (p.i. 28–35) stages. A typical pattern of CSF flow for naïve mice showed a delay before the tracer was detected and then a steady increase up to 60 min in the signal of the P40D800 tracer (Fig. 1B, Supplemental video 1). In contrast to the naïve group, all mice in the preonset, onset, peak and chronic groups showed minimal increase of tracer signal (Fig. 1B, Supplemental video. 2). All EAE groups demonstrated a statistically significant decline in the signal detected in the thoracic spine at 60 min as compared to the naïve group (Fig. 1E, Naïve: 10260 ± 3670, Preonset: 1234 ± 1019, *p* < 0.0001; Onset: 489 ± 315, *p* < 0.0001; Peak: 556 ± 316, *p* < 0.0001; Chronic: 1383 ± 518, *p* = 0.0003; one-way ANOVA). These results suggest that CSF flow within the SAS towards the sacral spinal cord is significantly impaired prior to EAE disease onset and that this impairment persists for the entire EAE disease course.

We previously demonstrated that after i.c.v-infusion in naïve mice, besides the apparent flow in the spinal SAS, CSF tracers also spread rapidly within the central canal to reach the sacral end of the spine [30]. In the current study, at the completion of dynamic tracking of P40D800 tracer signal at the thoracic spine at 60 min post-infusion, we also immediately took an image of the sacral region (Fig. 1C) which provides additional information on the CSF flow through the central canal. Although there is a tendency towards reduced tracer signals at preonset, onset and peak disease, no statistical differences from the naïve group were detected (preonset: 1034 ± 386, onset: 923 ± 902, peak: 491 ± 293, naïve: 2418 ± 1825, preonset vs. naïve *p* = 0.303, onset vs. naïve, *p* = 0.315; peak vs. naïve: *p* = 0.099; one-way ANOVA, Fig. 1F). Strikingly, however, tracer signals at the sacral spine of animals of chronic EAE were significantly stronger than naïve mice (5437 ± 3203, vs. naïve group *p* = 0.014; one-way ANOVA, Fig. 1F). These results suggest that at the chronic stage of EAE, CSF tracers flow efficiently to the sacral region. Most likely this flow occurs through the central canal, given that tracer signals measured at the thoracic region SAS were still significantly reduced at the chronic stage as compared to the naïve group.

**Fig. 2:**
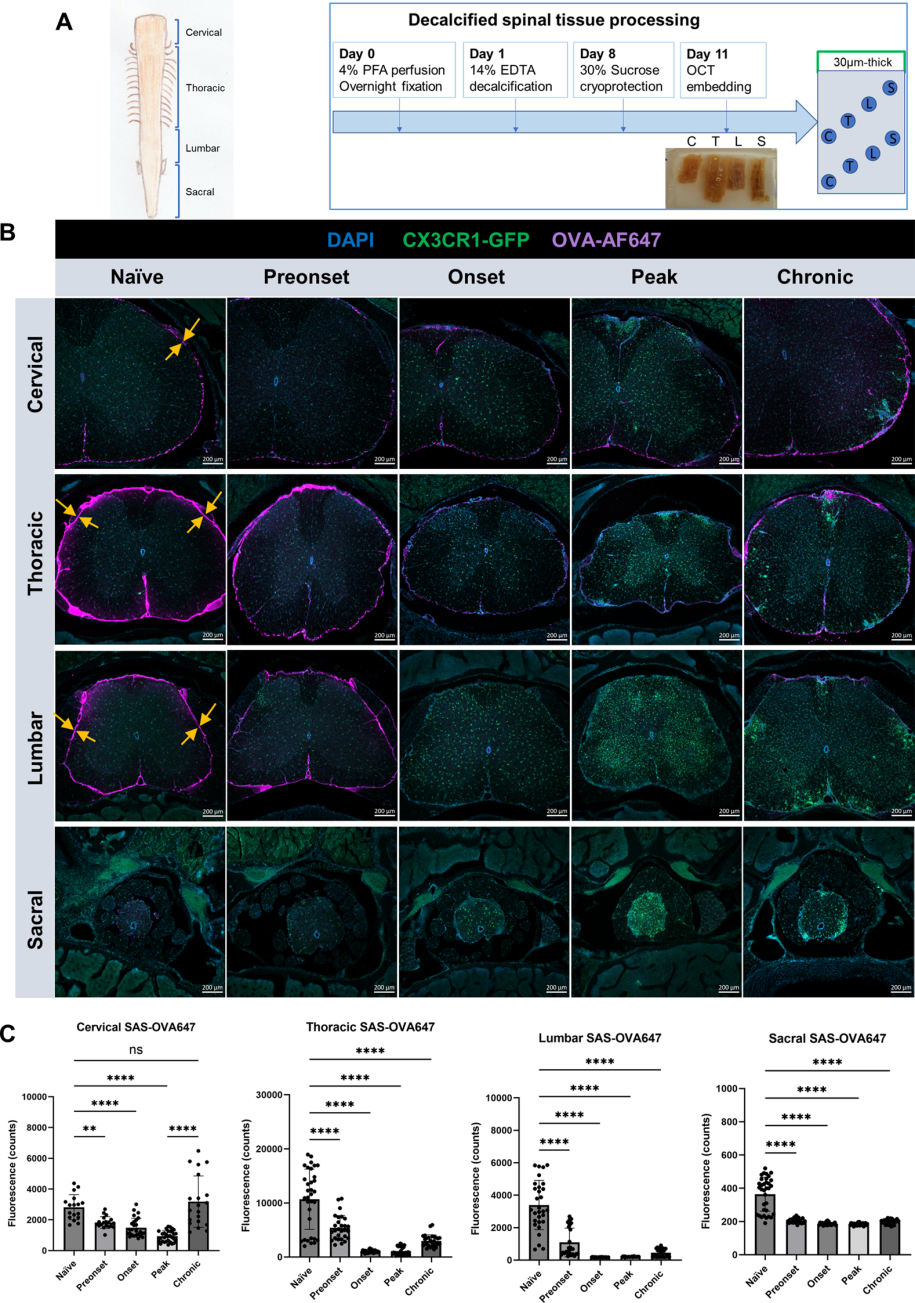
Assessments of OVA-AF647 signals in decalcified spinal columns confirm CSF flow impairment. **(A)** Schematics of spinal cord segments (left) and decalcification protocol with tissue processing (right). Decalcified spinal column was cut into four segments (C: cervical, T: thoracic, L: lumbar, S: sacral) and further into 30 μm cryosections. **(B)** Representative overview images of OVA-AF647 distribution in the SAS of spinal cord of naïve and EAE mice at preonset, onset, peak and chronic stages. Opposing arrows mark the interface between nerve roots and the spinal cord parenchyma. Scale bars: 200 μm. **(C**) Quantification of OVA-AF647 signal in the SAS of each spinal cord segment (one-way ANOVA with Dunnett’s post hoc test., ns, no significant difference; **, *p* < 0.01; ****, *p* < 0.0001)

Next, we analyzed the distribution of the co-infused OVA-AF647 tracer within the SAS and central canal, which not only allowed us to confirm the in vivo NIR imaging findings, but also provided more detailed information regarding each spinal cord segment. Spinal columns were decalcified and separated into cervical, thoracic, lumbar and sacral segments and cut into 30 μm-thick tissue sections (Fig. 2A). In order to precisely locate tracers within the SAS of the spinal cord, we first performed E-cadherin staining to mark the arachnoid barrier layer [36, 37] in the decalcified spinal cord tissue (Supplemental Fig. 1A-D). After infusion into naïve mice, confocal imaging confirmed that OVA-AF647 tracer was predominantly confined to the SAS (Supplemental Fig. 1E). OVA-AF647 tracer was distributed on the surface of the spinal cord and along the interface between nerve roots and the spinal cord parenchyma (Fig. 2B). In naïve animals (Fig. 2B, C), the strongest OVA-AF647 signals were found in the thoracic spinal cord (10677 ± 5569), while cervical (2820 ± 817) and lumbar segments (3397 ± 1518) showed less signal. Only minimal signals were found in the sacral spine (364 ± 102). In the EAE groups at preonset (thoracic: 5384 ± 2281, lumbar: 1090 ± 882, sacral: 205 ± 14, vs. naïve: *p* < 0.0001, cervical: 1823 ± 381, vs. naïve: *p* < 0.001, one-way ANOVA), onset (cervical: 1492 ± 608, thoracic: 1051 ± 250, lumbar: 179 ± 8, sacral: 186 ± 9, vs. naïve: *p* < 0.0001, one-way ANOVA ) and peak (cervical: 935 ± 358, thoracic: 1109 ± 750, lumbar: 203 ± 18, sacral: 183 ± 3, vs. naïve: *p* < 0.0001, one-way ANOVA), all four spinal cord segments showed significantly reduced OVA-AF647 signals within the SAS as compared to their naïve counterparts. Interestingly, the cervical spinal cord of EAE mice at the chronic stage (3187 ± 1669) showed similar levels of OVA-AF647 signals as compared to that of naïve mice (*p* = 0.692), whereas the thoracic (2984 ± 1116), lumbar (459 ± 248) and sacral spinal cord (200 ± 14) still showed significantly reduced signals as compared to the naïve group (*p* < 0.0001, one-way ANOVA). In summary, the results from decalcified spinal tissue confirmed the above NIR imaging data, indicating that there is significant impairment of CSF flow along the entire spinal cord at preonset, onset and peak EAE stages. Furthermore, at the chronic stage of EAE, there is recovery of CSF distribution of OVA-AF647 to the cervical spinal cord.

### CSF flow changes along the spinal cord central canal during EAE

**Fig. 3:**
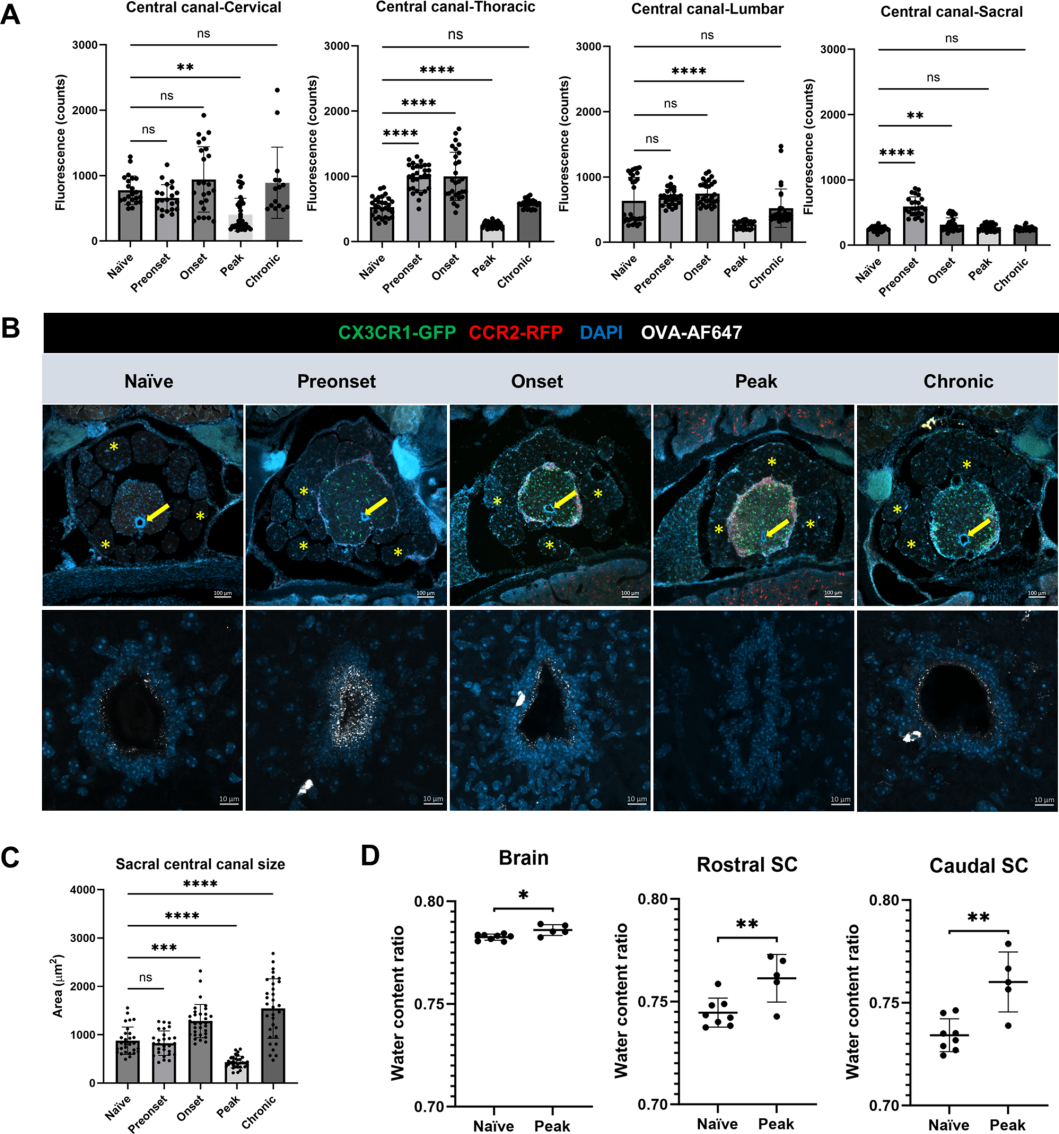
CSF flow along the central canal fluctuates during EAE development. **(A)** Quantification of OVA-AF647 distribution inside the central canal (data are pooled from 3 animals per condition). **(B)** Upper panel, overview of the sacral spinal cords exhibiting signs of edema (merged nerve roots) at EAE peak (asterisk: nerve roots, arrow: central canal). Lower panel, confocal images of sacral spinal cords show OVA-AF647 distribution on the wall of central canal. At EAE peak, no OVA-AF647 signal is visible and central canal appears collapsed. Scale bars: 100 μm (upper panels), 10 μm (lower pannels). **(C)** Quantification of the size of central canal. **(D)** Quantification of the water content ratio (*n* = 8 naïve brain, rostral SC and caudal SC., *n* = 5 peak brain, rostral SC and caudal SC). SC, spinal cord. Statistics with one-way ANOVA with Dunnett’s post hoc test (ns, no statistical difference; *, *p* < 0.1, **, *p* < 0.01; ***, *p* < 0.001; ****, *p* < 0.0001)

Quantification of OVA-AF647 signals within the central canal (Fig. 3A) revealed that a significant reduction only occurs at the EAE peak stage (cervical: 402 ± 252, thoracic: 256 ± 43, lumbar: 272 ± 57), as compared to the naïve group (cervical: 851 ± 1546, thoracic: 530 ± 151, lumbar: 636 ± 356). Nevertheless, at the chronic stage, OVA-AF647 signals (cervical: 889 ± 545, thoracic: 581 ± 66, lumbar: 521 ± 293) returned to similar levels as naïve group. Interestingly, in the thoracic region at the preonset (989 ± 203) and onset (999 ± 369) stages of EAE we found significantly increased OVA-AF647 signal inside the central canal. It must be noted that with the same image settings of the epifluorescence microscope, the sacral spinal cord central canal showed barely detectable OVA-AF647 signal even in naïve mice (245 ± 33). While this made it possible to detect significant increases of signal (e.g. preonset: 593 ± 145, *p* < 0.0001 vs. naïve; onset: 312 ± 105, *p* < 0.01 vs. naïve; one-way ANOVA), it precluded any measurements of reductions of signal in the central canal. Therefore, we had a close examination of the sacral spinal cord using confocal microscopy (Fig. 3B). Strikingly, at the EAE peak stage, the central canal at the sacral spinal cord appeared collapsed on sections losing its characteristic oval shape, and no detectable OVA-AF647 was found. Additionally, spaces between spinal nerve roots of the cauda equina were no longer apparent and appeared closely attached to the parenchymal surface (Fig. 3B). Accordingly, quantification of the area of the central canal at the sacral spinal cord revealed that the central canal was expanded at EAE onset and collapsed at the peak of EAE, while at the chronic stage of EAE the central canal was significantly expanded compared to naïve mice (naïve: 875 ± 282; onset: 1281 ± 343, vs. naïve: *p* = 0.0001; peak: 433 ± 122, vs. naïve: *p* < 0.0001; chronic: 1540 ± 615, vs. naïve: *p* < 0.0001; one-way ANOVA, Fig. 3C).

**Fig. 4:**
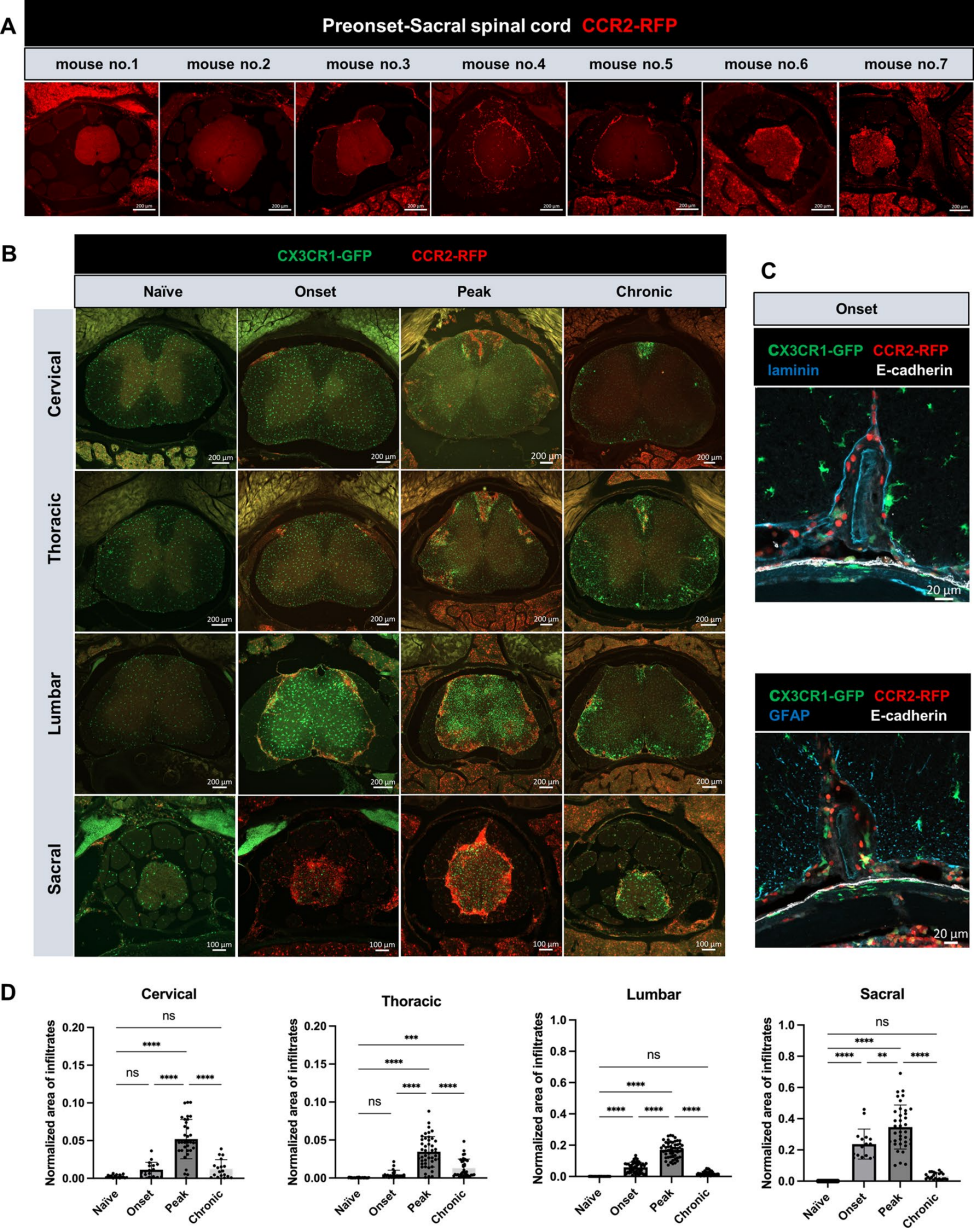
Caudal-rostral development of CCR2^+^ infiltrates during EAE. **(A)** Representative overview images of sacral spinal cord showing varying degrees of CCR2^+^ infiltrates in 7 mice from preonset group. Scale bars: 200 μm. **(B)** CCR2^+^ cell distribution on each of the four segments of spinal cord of naïve, onset, peak and chronic EAE mice. **(C)** Confocal images of lumbar spinal cord with Laminin/E-cadherin and GFAP/E-cadherin co-immunostaining showing CCR2^+^ infiltrates first appear in the SAS of the spinal cord. **(D)** Quantification of CCR2^+^ cells covered area normalized to the area of each spinal cord section. Each data point is one measurement of one image for a specific spinal cord segment. Data are pooled from 3–6 mice per experimental group, presented as mean ± SD (one-way ANOVA with Dunnett’s post hoc test, ns, no significant difference; **, *p* < 0.01; ***, *p* < 0.001; ****, *p* < 0.0001)

**Fig. 5:**
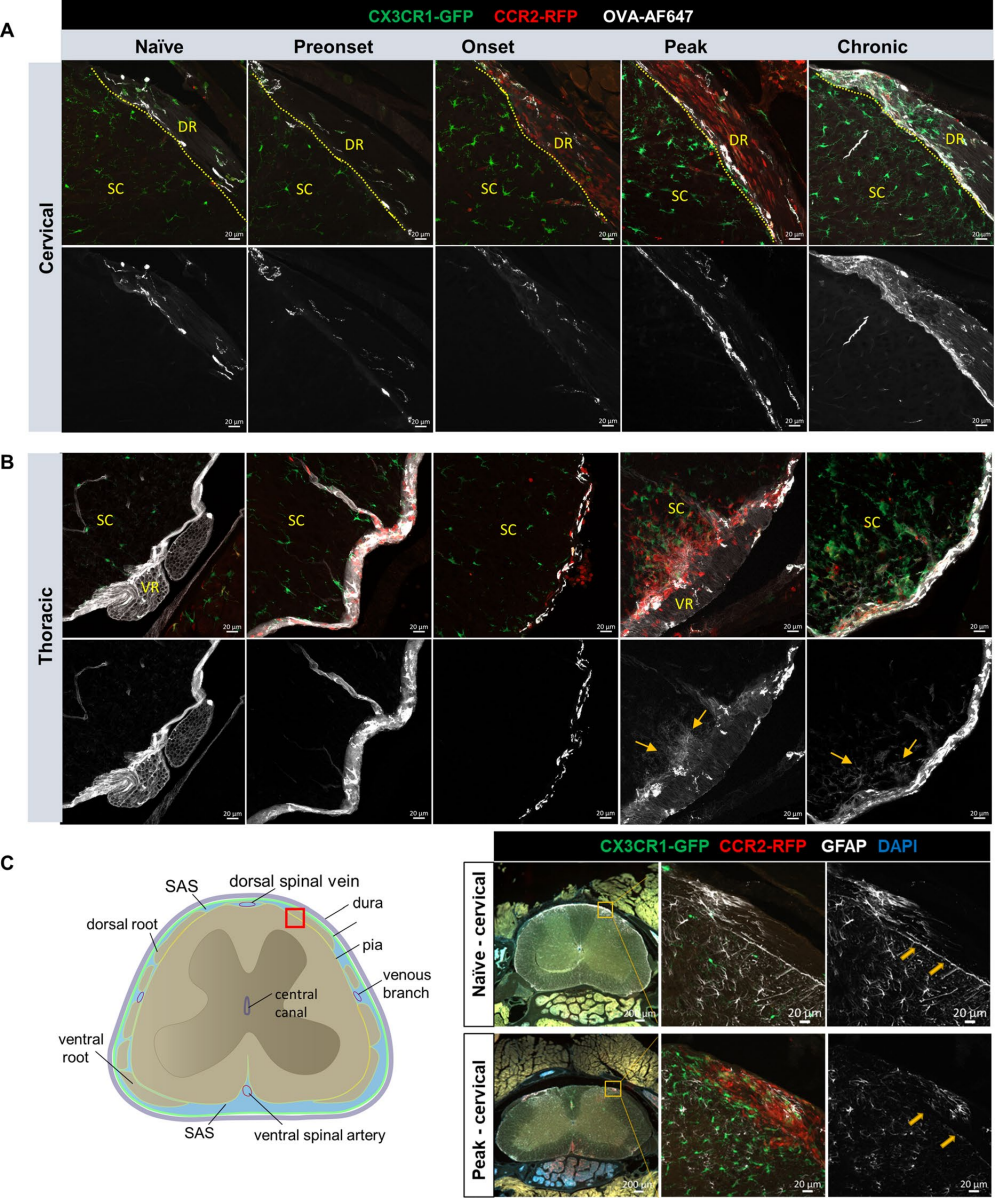
OVA-AF647 tracer not found in the spinal cord parenchyma at early stage of EAE. **(A)** Confocal images of OVA-AF647 distribution on the dorsal aspect of cervical spinal cords, where OVA-AF647 tracer signals are seeing at the interface (dotted line) between dorsal root (DR) and spinal cord (SC), but no obvious OVA-AF647 is visible in the parenchyma of spinal cord, except for occasional perivascular distribution, e.g. at chronic stage. Scale bars: 20 μm **(B)** Confocal images of OVA-AF647 distribution on the ventral aspect of thoracic spinal cords. In naïve, preonset and onset groups, OVA-AF647 signals are mostly found on the surface of the spinal cord, and in perivascular spaces, but not inside the parenchyma. From the mouse at EAE peak and chronic stage, clear signals of OVA-AF647 are found inside the parenchyma, where abundant CCR2^+^ infiltrates are also found. Scale bars: 20 μm **(C)** Representative images of GFAP staining from cervical spinal cord show continuous (naïve) and discontinuous GFAP immunoreactivities (arrows, peak EAE) at the interface between the spinal cord parenchyma and dorsal roots (red box on the schematic)

**Fig. 6:**
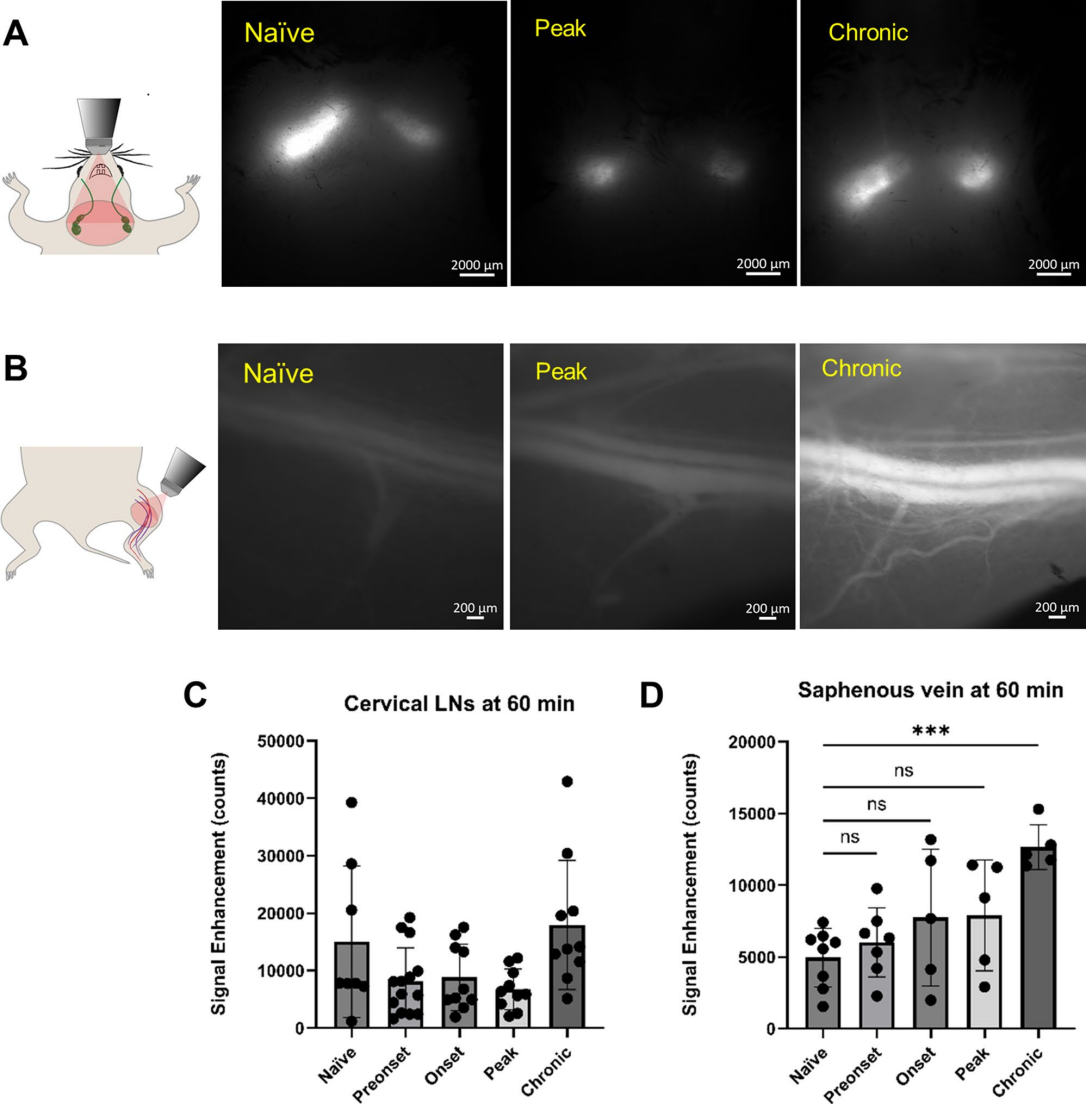
CSF tracer clearance to superficial cervical lymph nodes (scLNs) and the systemic circulation is maintained during EAE. **(A)** Schematic and representative picture of P40D800 tracer within the scLNs at 60 min post i.c.v-infusion. **(B)** Schematic and representative pictures of P40D800 tracer within the saphenous vein 60 min post i.c.v-infusion. **(C)** Quantification of fluorescence signal enhancement of P40D800 tracer within the scLNs, no statistical differences are found as compared to naïve group (signals from left and right scLNs are pooled together). **(D)** Quantification of fluorescence signal enhancement of P40D800 tracer within the saphenous vein. Statistics with one-way ANOVA with Dunnett’s post hoc test, no significant difference; ***, *p* < 0.001

**Fig. 7:**
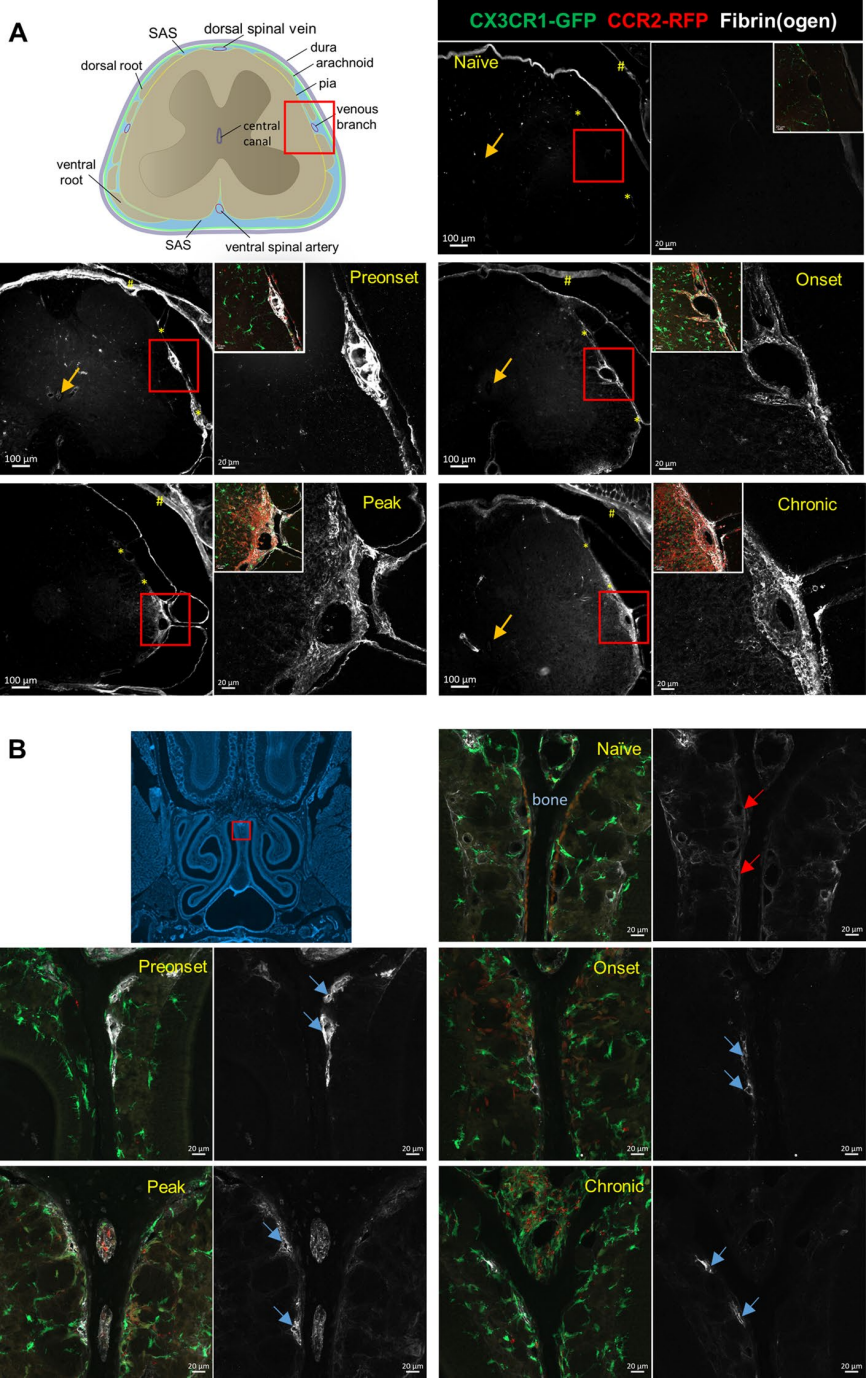
Fibrin(ogen) detected in spinal leptomeninges prior to EAE disease onset and the entire disease course. **(A)** Fibrin(ogen) staining is positive around the lateral venous branch and at the interface (*) between the spinal cord parenchyma and dorsal roots of mice in all conditions, except for the naïve mice. Note that dura mater (#) shows positive fibrin(ogen) staining due to fenestrated blood vessels inside dura mater. For each condition, left panel shows an overview picture and right panel shows the confocal picture of area inside the red boxes. Inserts on the right panels are overlay pictures of the same region. Arrows mark negative fibrinogen staining inside the central canal in all conditions. **(B)** Positive fibrin(ogen) immunoreactivies are seen in the lumen of vessels (blue arrows) against the nasal septum bone (dark area between the orange lines shown in naïve condition) in all EAE conditions, but not in Naïve condition (red arrows). Scale bars: 20 μm

**Fig. 8:**
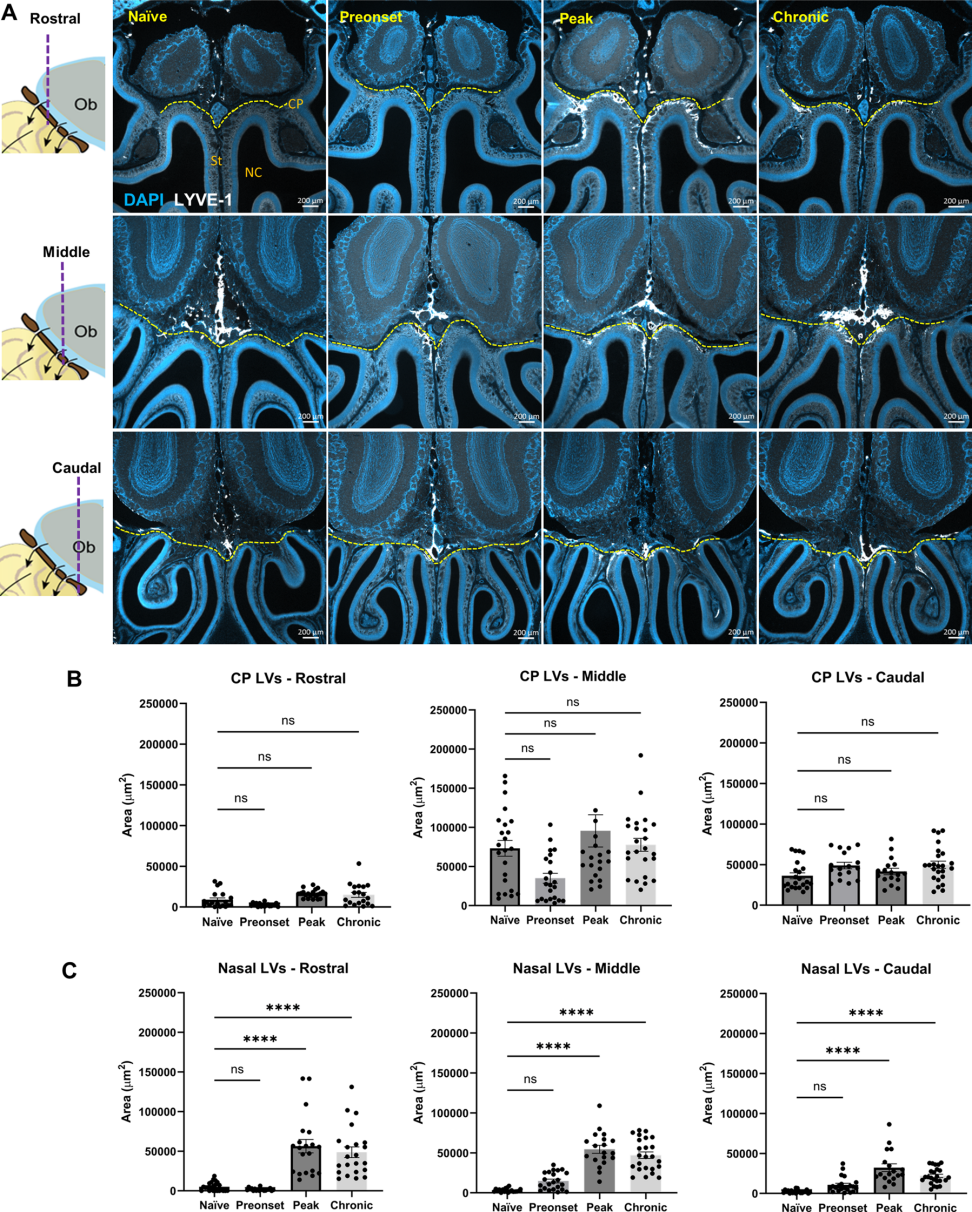
Significantly enlarged LYVE-1^+^ lymphatic vessel covered area. **(A)** Schematics on the left showing the rostral, middle and caudal olfactory bulb (Ob) region that representative images of LYVE-1 and DAPI staining on decalcified coronal sections for each experimental group were taken. Yellow dashed line marks the location of cribriform plate (CP). St, septum., NC, nasal cavity. **(B)** Quantification of LYVE-1^+^ area above/through the cribriform plate (CP-LVs) at rostral, middle and caudal olfactory region. **(C)** Quantification of LYVE-1^+^ area below the cribriform plate (Nasal-LVs) at rostral, middle and caudal olfactory region. Data are pooled from 3 mice per experimental group, presented as mean ± SD (one-way ANOVA with Dunnett’s post hoc test; ns, no significant difference; ****, *p* < 0.0001)

We speculated that the size reduction of the central canal and merging of nerve roots at the sacral spinal cord were signs of tissue swelling. Therefore, we performed wet-dry measurements of parenchymal water content at the EAE peak stage compared to naïve mice (Fig. 3D) Our data showed that both rostral and caudal spinal cord segments of EAE mice contained significantly higher water content ratios than those of naïve mice (rostral spinal cord, EAE: 0.761 ± 0.011 vs. naïve: 0.745 ± 0.007, *p* = 0.0073; caudal spinal cord, EAE: 0.760 ± 0.015 vs. naïve: 0.734 ± 0.008, *p* = 0.0015; two-tailed students’ *t test*). Indicative of edema throughout the CNS, the brains from EAE mice also showed slightly higher water content ratio than that of naïve mice (EAE: 0.786 ± 0.003 vs. naïve: 0.783 ± 0.001, *p* = 0.011; two-tailed students’ *t test*).

Taken together, these data demonstrate that CSF flow along the central canal was blocked at EAE peak phase likely due to a compression of the central canal resulting from spinal cord tissue edema. At EAE chronic stage, CSF flow through the central canal was re-established to similar levels as the naïve group.

### Development and resolution of infiltrating CCR2^+^ cells are associated with changes in spinal CSF flow

Next, we aimed to investigate if the CSF flow impairment along the SAS of spinal cord was associated with the presence of CCR2^+^ immune cell infiltrates in the leptomeninges. Coinciding with the impairment of CSF flow, beginning at the preonset stage, 6 out of 7 of the mice in this group presented with CCR2^+^ cells at varying levels of infiltration, confined mostly to the sacral spinal cord segment (Fig. 4A). At the same time, the cervical spinal cord segments showed almost no CCR2^+^ infiltrates, while the thoracic spinal cord showed minimal CCR2^+^ cell infiltrates and the lumbar spinal cord had detectable CCR2^+^ cells only in some mice (Supplemental Fig. 2A). At EAE onset, CCR2^+^ cells were visible at all segments of the spinal cord. In the naïve group, CCR2^+^ cells were not apparent in the SAS at any segment of the spinal cords (Fig. 4B).

E-cadherin co-immunostaining with laminin or GFAP indicated that these cells were mainly distributed in the leptomeninges of the cervical, thoracic and lumbar spinal cord (Fig. 4C) consistent with a recent report [11]. In addition, at the onset stage of disease, these cells were also found within the spinal cord parenchyma of the sacral spinal cord. With disease progression to peak phase, CCR2^+^ infiltrates were found in the leptomeninges and parenchyma of all spinal cord segments. We quantified the CCR2^+^ area of cervical, thoracic, lumbar and sacral segments of mice at three disease stages and in naïve mice (Fig. 4D). At EAE onset, only lumbar and sacral segments showed significantly higher CCR2^+^ infiltrates as compared to naïve mice (lumbar onset: 0.059 ± 0.033 vs. lumbar naïve: 0.0004 ± 0.0004, *p* < 0.0001; sacral onset: 0.238 ± 0.096 vs. sacral naïve: 0.0003 ± 0.0002, *p* < 0.0001; one-way ANOVA). At EAE peak stage, all four segments showed significantly larger CCR2^+^ areas compared to naïve mice (cervical peak: 0.052 ± 0.026 vs. cervical naïve: 0.003 ± 0.002, *p* < 0.0001; thoracic peak: 0.035 ± 0.02 vs. thoracic naïve: 0.0004 ± 0.0003, *p* < 0.0001; lumbar peak: 0.171 ± 0.051 vs. lumbar naive: 0.004 ± 0.0004, *p* < 0.0001; sacral peak: 0.346 ± 0.141 vs. sacral naïve: 0.0003 ± 0.0002, *p* < 0.0001; one-way ANOVA). Interestingly, at EAE chronic stage, only the thoracic region still had significant CCR2^+^ cell coverage vs. the naïve group (chronic thoracic: 0.013 ± 0.012 vs. thoracic naive: 0.0004 ± 0.0003, *p* = 0.0004; one-way ANOVA).

These results suggest that infiltration of CCR2^+^ cells are first apparent at the sacral leptomeninges at the preonset stage, concurrent with the first signs of CSF flow impairment. At the same timepoint the cervical and thoracic spinal cord showed very minimal CCR2^+^ infiltrates. We conclude that spinal CSF flow impairment occurs prior to the appearance of significant immune cell infiltrates in the spinal SAS.

### Impairment of spinal CSF flow and early presence of CCR2^+^ infiltrates at the sacral spinal cord are not dependent of the site of EAE induction

Since we induced EAE by subcutaneous injections of MOG_35 − 55_/CFA (40 µl at each flank and 20 µl near the tail base), we next investigated if the CCR2^+^ cells found at the sacral spinal cord at preonset and the significant impairment in CSF flow towards the sacral spinal cord were potentially due to a local inflammation at the injection sites. Thus, in a group of 5 mice (two independent experiments), we instead induced EAE by subcutaneous injection of 50 µl of MOG_35 − 55_/CFA at the shoulder of each upper limb (shoulder control). All mice showed similar disease development compared with the standard protocol, starting with a limp tail at p.i.11 until reaching the peak disease between p.i.14 and p.i.17. We also performed NIR imaging at the EAE peak stage by infusing a mixture of P40D800 and OVA-AF647 into the lateral ventricle of 4 of these mice. These mice also showed impaired CSF flow similar to the mice that had been immunized near the tail base (Supplemental Fig. 3A). In addition, these mice demonstrated similar histopathological features at EAE peak compared to those that have been subjected to the routine EAE immunization protocol (Supplemental Fig. 3B). These data indicate that the impaired CSF flow and presence of CCR2^+^ infiltrating cells in the sacral leptomeninges are not due to a disease induction close to the sacral spine.

### CSF flow impairment is not dependent on the size of i.c.v-infused tracer

Since both P40D800 and OVA-AF647 tracers are around 40 kDa in molecular weight, we further assessed the distribution of a larger tracer (106 nm diameter DiD-labeled liposomes) to test whether the measured loss of flow in the spine could be a by-product of a potential loss of tracer through compromised CNS barriers. We sacrificed mice for decalcification at 2 h instead of 1 h post-infusion to allow more time for potential tracer distribution throughout the SAS towards the sacral spinal cord. Longitudinal sections of the spinal cord showed clear liposome signals at the sacral spinal cord SAS of naïve mice; however, liposomes were confined to the cervical and thoracic spinal cord of mice at EAE peak (Supplemental Fig. 4A, B), consistent with the findings from OVA-AF647 tracer (Fig. 2). In addition, confocal images showed that even at peak disease the E-cadherin positive arachnoid barrier layer surrounding the spine appeared morphologically intact, and liposomes were not found within the dura mater (Supplemental Fig. 4C, D).

### CSF flow impairment precedes the breakdown of glia limitans superficialis

The surface of brain and spinal cord is covered by the glia limitans superficialis, lying directly under the pia mater, composed of astrocyte foot processes and the parenchymal basement membrane [38, 39]. Low molecular weight tracers from CSF can pass the pia and glia limitans through diffusion to enter CNS parenchyma [40], however, the glia limitans poses a second barrier for the migration of leukocytes and monocytes into the CNS parenchyma [41, 42]. We speculated that 40 kDa tracers in CSF would more readily access the spinal cord parenchyma during EAE, a diffusive process that would be enhanced upon breakdown of glia limitans superficialis and during reduced CSF flow. This phenomenon could also potentially contribute to less distribution of CSF tracers to the SAS of the sacral spinal cord. Thus, we closely examined OVA-AF647 signals using confocal microscopy at the cervical and thoracic spinal cord (Fig. 5A, B). For the cervical spinal cord, we focused on the interface between dorsal spinal roots and spinal cord parenchyma, where abundant CCR2^+^ cells were often found at onset and peak stage. Our data showed that there is no clear OVA-AF647 signal in the parenchyma of the cervical spinal cord except for occasional perivascular distribution (Fig. 5A). For the thoracic spinal cord, assessments of the ventral aspect of the spinal cord revealed that at preonset and onset stages, OVA-AF647 signals were largely confined to the leptomeninges of the spinal cord, except for occasional perivascular distribution. At EAE peak and chronic stages, clear signals of OVA-AF647 were found inside the parenchyma where abundant CCR2^+^ infiltrates were also found (Fig. 5B).

We next aimed to confirm the integrity of the glia limitans superficialis at the peak stage of EAE by immunostainings for GFAP (glial fibrillary acidic protein, revealing astrocyte endfeet) and laminin (to assess the parenchymal basement membrane) [43]. An overview of GFAP staining demonstrated a clear outline along the edge of the spinal cord parenchyma indicating positive staining of astrocyte endfeet in naïve mice. However, at peak stage only diffuse GFAP immunoreactivity could be observed at the dorsal root and spinal cord interface (Supplemental Fig. 5A, B). The discontinuity of GFAP staining was strongly associated with CCR2^+^ cell infiltrates. We also observed a clear discontinuous pattern of GFAP staining at the cervical spinal cord (Fig. 5C) at the interface between the dorsal root and the spinal cord, indicating that the astrocyte endfeet at this location were compromised at EAE peak stage. We further confirmed the breakdown of the glia limitans superficialis at the lumbar spinal cord during EAE peak by laminin staining. Overview images of the spinal cord showed that laminin was clearly visible at the cervical spinal cord surface, while at the lumbar spinal cord of EAE mice it was nearly undetectable (Supplemental Fig. 5C). Confocal examinations showed clear laminin staining at the lumbar spinal cord-dorsal root interface of naïve mice; however, this interface was packed with CCR2^+^ infiltrates without detectable laminin staining at EAE peak. Taken together, our data suggest that impairment of spinal CSF flow occurs prior to the breakdown of glia limitans superficialis.

### Drainage of CSF to cervical lymphatics remains functional during EAE and overall CSF turnover is enhanced at the chronic stage of EAE

In the same groups of mice in which dynamic tracking of CSF tracer flow along the thoracic spine was performed (Fig. 1), we also acquired images of tracer clearance to the superficial cervical lymph nodes (scLNs) and the saphenous vein at 60 min to provide measures of CSF lymphatic efflux [22]. We observed similar levels of P40D800 tracer signal within the lymph nodes in all groups (Fig. 6A, C), suggesting that cranial lymphatic outflow pathways towards the scLNs remained functional despite the impairment in CSF flow seen at the spine.

Measures of tracer recovery in the systemic blood circulation after CSF tracer infusion provide an overall assessment of lymphatic clearance routes [22], including the pathways to both superficial and deep cervical lymph nodes and spinal efflux. We assessed the signals in the saphenous vein after 60 min and found no statistical differences at pre-onset, onset and peak stages as compared to naïve group. However, saphenous vein tracer signals were significantly enhanced at EAE chronic stage (Fig. 6B, D). These results suggest that during the development of EAE until the peak stage comparable amounts of i.c.v-infused CSF tracer were returned to the systemic circulation as in naïve mice, but that higher amounts were cleared out of the CNS at the chronic stage.

### Leptomeningeal vascular leakage occurs prior to EAE onset and manifests as an accumulation of fibrin(ogen) within the subarachnoid space across the course of EAE

Previously, it has been shown that upon BBB breakdown, fibrinogen accumulates in the CNS and is converted into insoluble fibrin [44]. Consequently, we performed immunofluorescence staining against fibrin(ogen) on decalcified spinal sections and compared the fibrin(ogen) immunoreactivity around the large venous branch on the lateral aspect of the lumbar spinal cord. In contrast to naïve mice where fibrinogen immunoreactivity was negative (Fig. 7A), all EAE animals showed positive fibrin(ogen) staining at this location. At the preonset phase, fibrin-mesh networks with numerous embedded CCR2^+^ cells were apparent in the leptomeninges of the lumbar spinal cord around the dorsal spinal vein (Supplemental Video 3). With EAE disease progression, widespread fibrin(ogen) deposits appeared in the leptomeninges, as well as in the parenchyma of the spinal cord at clinical peak and chronic stages. Notably, clear fibrinogen staining within the central canal was not detectable at any disease stage (Fig. 7A). These data demonstrate that during EAE disease development, deposits of fibrinogen/fibrin within the SAS of spinal cord occur in parallel with the spinal CSF flow impairment.

### Nasal lymphatics drain fibrin(ogen) and upregulate LYVE-1 expression during the course of EAE

Previous studies from our group and others have shown that the majority of cranial CSF drains into the lymphatic vessels (LVs) which pass through the cribriform plate (CP) and are situated in the nasal submucosa [45–47]. A prior study has shown that during peak EAE the LVs near the cribriform plate undergo lymphangiogenesis and may contribute to the drainage of CNS-derived antigens and immune cells [48]. Thus, it is likely that stable levels of CSF outflow to cervical LNs throughout the EAE disease course are facilitated by functional nasal LVs. Thus, we tested if nasal LVs are capable of draining fibrinogen during EAE. As shown in our previous study, nasal lymphatics can be readily seen closely distributed against the nasal septum bone [45]. While there is no evident fibrin(ogen) immunoreactivity in naïve mice near the septum (Fig. 7B), during all phases of EAE, we could identify several vessel-like structures at this location that demonstrated strong fibrin(ogen) staining.

Next, we evaluated whether alterations in lymphatic vessel endothelial hyaluronan receptor 1 (LYVE-1) positive lymphatic vessel covered area could be found in the LVs crossing the cribriform plate, which might suggest that lymphangiogenesis is occurring in these vessels [48]. We performed this analysis on LVs above (including those passing through) and below the CP (denoted as nasal LVs in this study) at multiple EAE disease phases (Fig. 8A). To our surprise, we did not find a significant change in the LYVE-1^+^ area above the CP at any given EAE disease stage (Fig. 8B), however, a significantly larger LYVE-1^+^ area was found below the CP at EAE peak and chronic stage compared to naïve conditions (Fig. 8C). These data further highlight the potential role of nasal LVs in draining fibrinogen and their plasticity to neuroinflammatory environments.

## Discussion

The MOG_35 − 55_ induced active EAE mouse model is known to present with leptomeningeal immune cell infiltrates, leptomeningeal vessel leakage and glia limitans breakdown, yet the time course of these events is not completely clear [41, 42, 49]. In this study, we examined the time course of changes in spinal CSF flow dynamics in relation to the leptomeningeal pathophysiology during active EAE. Our study demonstrated that a dramatic impairment of CSF flow along the SAS of the spinal cord was initiated at the preonset stage and persisted until the peak stage. The spinal CSF flow impairment occurred in parallel with fibrin(ogen) leakage in the SAS but preceded the accumulation of peripheral immune cells and glia limitans superficialis breakdown at the spinal leptomeninges. This data suggests that imaging changes in CSF flow dynamics may serve as a sensitive marker for leptomeningeal pathology.

A previous study by Fournier et al. utilizing gadolinium contrast agent (Gd-DOTA) enhanced MRI and ex vivo imaging of intra-cisterna magna injected indocyanine green in a different EAE model (Proteolipid protein, PLP immunization in SJL/J mice) showed that diffusion of low molecular weight tracers to the spinal cord parenchyma was significantly reduced in EAE mice with clinical disease [50]. Due to the lack of data on tracer localization at the histological level in this previous study, we cannot infer whether the observation of reduced parenchymal diffusion of small tracers is due to the reduced CSF flow within the spinal SAS. However, it is conceivable that a significant reduction of CSF flow in the SAS will lead to less CSF influx into the parenchyma at least when the glia limitans superficialis is intact. Moreover, Fournier et al. did not observe CSF influx changes at the preonset stage. Interestingly, Lepore et al. using a PLP-induced EAE model in SJL/J mice demonstrated that ventricular enlargement indeed took place at the preonset stage [51] and a later study from the same group showed that the size of the ventricles returns to normal levels upon EAE remission [52]. These authors also observed an alteration in the T2 relaxation times of the CSF, which was interpreted to occur due to altered CSF composition or increased production of CSF. Previous studies have suggested a strong correlation between ventricular enlargement and CSF production in the case of idiopathic normal pressure hydrocephalus [53, 54]. Our data showed that the size of the sacral central canal varied from enlargement at the onset stage to reduction at the peak stage and rebound at the chronic stage. These changes were accompanied by a significant increase of CSF tracers inside the spinal cord central canal at the preonset/onset stages, a significant reduction at peak stage and a return to normal levels at chronic stage (Fig. 3A, C). Whether these changes in central canal are due to changes in CSF production or occur for other reasons (e.g. spinal cord edema or blocked CSF flow along SAS) is currently unknown. Nevertheless, these results may shed light on central canal pathology in MS patients. Some MS patients have been shown to develop non-communicating syringomyelia (enlargement of the central canal) and syrinx formation [55–57]. Even though the exact mechanism of syrinx formation is unclear, it has been hypothesized that accumulation of fluid in the syrinx is due to abnormal CSF drainage from the central canal [58–60].

Taken together, these data support the notion that leptomeningeal inflammation could disrupt the circulation of CSF. In a PLP-induced EAE model in SJL/J mice, Millward et al. reported meningeal infiltration of macrophages/myeloid cells at d5 p.i. which was earlier than the statistical increase in the ventricular size at d8 p.i. By d11 p.i., the significantly increased ventricular size was accompanied by extensive histopathology [52]. In our study, the significantly reduced spinal CSF flow at the rostral spinal cord preceded immune cell infiltration into the CNS and continued CSF flow impairment was accompanied by increased immune cell accumulation in the spinal leptomeninges. Interestingly, a partial recovery of CSF flow at the chronic stage was accompanied by significant reduction in the levels of immune cells as compared to the peak stage in all spinal cord segments. Bearing in mind that different measurements (e.g. MRI vs. histopathological analyses vs. NIR imaging) may have different sensitivity in detecting specific phenomenon, these correlated studies may give clues to important pathophysiological mechanisms. We suggest from our data that due to the lack of myeloid cells at the rostral spinal cord during preonset EAE, it is unlikely that CSF flow impairment at this early stage is initiated by the obstruction of SAS by immune cell infiltration but may arise from other factors.

BBB breakdown is one of the most important and early hallmarks of MS, which precedes the appearance of gadolinium-enhanced lesions in T1-weighted MRI and clinical symptoms of MS [61, 62]. Leptomeningeal blood vessels are present within the CSF-filled space bordered by arachnoid and pia mater cell layers. In contrast to endothelial cells of the BBB, leptomeningeal vessel endothelial cells constitutively express P-selectin, and display different tight junction molecular arrangements [63]. Breakdown of spinal cord leptomeningeal blood vessels has not been well studied during EAE, as the spinal cord is often extracted from the vertebral column and the leptomeninges easily damaged. Previous studies have shown that upon BBB breakdown, fibrinogen accumulates in the CNS and is converted into insoluble fibrin [44]. Fibrinogen is a blood coagulation 340 kDa plasma glycoprotein [64–66]. The conversion from soluble fibrinogen to insoluble fibrin results in its persistence in the CNS and additionally endows the exposure of an epitope from fibrin to bind to CD11b/CD18 integrin receptor on microglia cells, which has been shown to trigger cell activation and participate into lesion formation and neurodegeneration [44, 67]. Previously, it has been reported that fibrinogen and fibrinogen peptide A levels in CSF are significantly elevated in patients with primary progressive multiple sclerosis [68]. Using decalcified tissue sections, we found positive fibrinogen/fibrin immunoreactivity within the leptomeninges of the spinal cord throughout the entire EAE disease course. Due to the involvement of fibrinogen in multiple pathological aspects during EAE, it would be challenging to establish a direct link between the impaired CSF flow and fibrin deposits in the SAS. Nevertheless, given that these two events occur in parallel, and both take place prior to EAE onset, it is possible that leakage of fibrin(ogen) may contribute to the impairment of CSF flow in our EAE model.

Previous work from our group and others have demonstrated that i.c.v-infused CSF tracers clear within minutes from the cranium to cervical LNs through multiple pathways [22, 69, 70]. In contrast, in the spinal cord, i.c.v-infused CSF tracer flows rapidly down the central canal and more slowly through the spinal SAS towards the sacral spinal cord, eventually draining into lymphatics leading to the sacral and iliac LNs. The amount of CSF tracer draining to the iliac LNs was significantly less than the amount draining into cervical LNs indicating that the spinal route contributes to a smaller proportion of overall CSF clearance [30]. Based on these studies, one may reason that spinal CSF flow impairment during EAE disease course will lead to re-routing of CSF flow towards cranial clearance pathways. On the contrary, despite the significant impairment of CSF flow along spinal cord during EAE, CSF efflux to cervical lymph nodes 1 h post-infusion of CSF tracer was not significantly changed. This is consistent with the above-mentioned study by Fournier et al. [50] that the diffusion of Gd-DOTA and indocyanine green were normal in the brain and cerebellum of EAE animals. Whether the intact CSF efflux pathways from the cranium play a protective or a detrimental role in EAE is currently a matter of debate [11, 71, 72]. Lymphatic vessels near the cribriform plate have been shown to undergo lymphangiogenesis at EAE peak stage [48, 73], and presumably be responsible for the drainage of CD11c^+^ dendritic cells and CD4^+^ cells. Our study confirms that nasal LYVE-1^+^ lymphatic vessels cover a larger area at EAE peak stage. Additionally, there is no changes in LYVE-1^+^ lymphatics at preonset stage, but larger nasal LYVE-1^+^ lymphatics also exist at chronic stage. Hsu et al. applied a generic pharmacological inhibition of VEGFR3, showing that it delayed the onset of EAE and reduced the clinical score [48]. However, since this is not a specific inhibitor for cribriform plate lymphatic vessels, caution is needed to derive any conclusions on the impact of functional roles of these lymphatics play in EAE development. From the perspective of transporting infiltrated immune cells and CSF-borne fibrinogen out of the CNS, CSF drainage might assume a protective role. Functional adaptations of nasal lymphatics might be important for maintaining an effective CSF efflux route when other pathways are blocked. Nevertheless, it may also mask subtle changes in the overall status of CSF flow during leptomeningeal inflammation, arguing in favor of spinal CSF flow rather than CSF outflow to cervical LNs as being a more sensitive biomarker for leptomeningeal inflammation.

We acknowledge the following limitations in our study. Due to experimental restrictions, we were not able to perform longitudinal studies using NIR imaging to track dynamic changes of CSF flow within individual animals. Without a longitudinal study, the preonset stage was set as p.i.10, one day before the earliest day we detected disease onset in our model, which develops with nearly 100% disease incidence. While at p.i.10 all animals investigated showed impaired CSF flow, extending the study to an earlier timepoint would not guarantee that all mice would be at a similar disease state. Consequently, we cannot exclude the possibility that CSF flow impairment, at least in some animals, may occur even earlier than we have observed.

In conclusion, our study sheds light on the use of non-invasive imaging of spinal CSF flow as a sensitive biomarker to infer pathological changes within the spinal leptomeninges during neuroinflammation. Because leptomeningeal inflammation is often detected at the early stage of MS [74], developing a non-invasive imaging technique that can reflect pathological changes within the leptomeninges could be useful for early detection of disease onset. Previously, various MRI imaging techniques have been applied for the diagnosis and disease monitoring of MS [75] and for identifying novel biomarkers [76], however, the primary focus has been on imaging the CNS parenchyma, rather than the CSF per se. In MS and other neurological diseases, leptomeningeal enhancement can be visualized with MRI in T2/FLAIR sequences after the injection of gadolinium-based contrast agent [77, 78], however, the underlying pathology is unclear. Phase-contrast MRI has been applied to assess CSF flow dynamics along the spinal cord in amyotrophic lateral sclerosis (ALS) patients and revealed a reduction in CSF flow magnitude and increased flow velocities in the ALS patients as compared to a control cohort [79]. Such promising imaging paradigms could be employed to evaluate CSF flow dynamics in MS patients with the goal to develop non-invasive imaging biomarkers for early detection of pathological changes within the leptomeninges.

## Materials and methods

### Animals

Our study examined male and female *CCR2-RFP x CX3CR1‐GFP* mice [32], which were a kind gift from Dr. Israel F. Charo (UCSF, USA) and Dr. Richard Ransohoff (Boston, USA). Mice were bred in-house and kept in individually ventilated cages under specific pathogen-free conditions at a light-dark cycle of 13 h–11 h. Food and water were accessible ad libitum. EAE experiments were performed at 2–3 months of age and mice of both sexes were randomly assigned to experimental groups. *Prox1-GFP* [80] mice of 8 weeks of age were used for E-cadherin staining to mark the arachnoid layer. Our study examined male and female animals, and similar findings are reported for both sexes.

### Active experimental autoimmune encephalomyelitis (aEAE) induction and scoring

aEAE was induced by injecting an emulsion of myelin oligodendrocyte glycoprotein peptide_35–55_ (MOG_35-55_ peptide, Genscript), 200 µg per animal, in complete Freund’s adjuvant (CFA, prepared from Incomplete Freund’s Adjuvant, Santa Cruz Biotechnology, USA; supplemented with Mycobacterium Tuberculosis, Difco). In brief, 100 µl emulsion of MOG_35 − 55_ and CFA was injected subcutaneously in mouse flanks (80 µl) and at the tail base (20 µl) at day 0 under short isoflurane anesthesia. A second group of mice were injected subcutaneously with 100 µl emulsion of MOG_35 − 55_ and CFA into the shoulder area (50 µl on each side). Moreover, 400 ng PTX (List Biological Laboratories, Campbell, CA, USA) was applied intraperitoneally at day 0 and day 2. After immunization, mice were weighed and scored daily on a 3-point scale as described previously [81]. Wet chow was provided in a Petri dish as soon as the first mouse reached clinical onset. Four time points during aEAE were investigated: Preonset (clinical score 0, p.i.10), onset (clinical score 0.5, animals showing weight loss and a limp tail, p.i. 11-12), peak (clinical score 2, animals showing strong hindlimb paraparesis or paraplegia, p.i. 17-21) and chronic (clinical score 1.5 ± 0.4, animals presenting partial hindlimb paraparesis after prior paraplegia, p.i. 28-35). Naïve mice were not subject to immunization procedures.

### Tracer infusion into the lateral ventricle

Mice were anesthetized with an initial intraperitoneal injection of ketamine (80 mg/kg) and medetomidine (0.4 mg/kg) and anesthetized mice were shaved and depilated over the spine, sacrum, cervical lymph nodes and saphenous vein. For intracerebroventricular infusion (i.c.v), mice were given an additional injection of ketamine/medetomidine of 1/3 of the initial dose and fixed in a stereotactic frame (RWD, Mainz, Germany). The skull was thinned with a dental drill (F.S.T., North Vancouver, B.C., Canada) 0.92 mm lateral and 0.22 mm caudal from bregma. A 34G steel needle (Hamilton, Bonaduz, Switzerland) on a 10 µl gas tight syringe (Hamilton, Bonaduz, Switzerland) was inserted into the left lateral ventricle 2.35 mm ventral to the skull surface. NIR tracer of P40D800 [82] and DiD-labeled liposomes [34] were formulated in house. Alexa647-conjugated ovalbumin (OVA-AF647) was purchased from Thermo Fisher Scientific (Waltham, MA, USA). Infusion of 3 µl tracers (one tracer type alone, or P40D800 mixed with either liposomes or OVA-AF647) at a rate of 0.5 µl/min was performed with a syringe pump (Stoelting, Wood Dale, IL, USA). The needle was left in place for 2 min before being retracted slowly to avoid backflow. The skull was closed with bone wax (Ethicon, Somerville, NJ) and the skin wound was closed with tissue glue (Vetbond™, Fisher Scientific, Reinach, Switzerland).

### Near-infrared (NIR) imaging

Immediately after i.c.v tracer infusion, mice were quickly positioned under a Zeiss AxioZoom.V16 microscope, equipped with a Prime BSI Scientific sCMOS camera, (Teledyne Photometrics, Tucson, AZ, USA), a light-emitting diode illumination system pE-4000 (CoolLED Ltd, Andover, UK) and ZEN 2.6 software (Carl Zeiss, Feldbach, Switzerland). Mice were placed on a heating pad for a constant body temperature of 37 °C and the spine centered under the objective. A rectal temperature probe was inserted, a pulse oximeter fixed to the paw and a nosecone with > 90% O_2_ attached to the snout. Vital parameters were controlled on a Somnosuite^®^ rodent anesthesia machine (Kent Scientific, Torrington, CT, USA). Dynamic intravital through-skin NIR imaging above the highest raised point of thoracic vertebrae (roughly between vertebral T10 and T12), was initiated within 10 min and performed up to 60 min post infusion (illustrated in Fig. 1A). Images were taken every 30 s at an exposure time of 500 ms (excitation at 740 nm, ICG filter for P40D800 tracer) at 12x zoom. Directly after the dynamic imaging, pictures of the sacrum (zoom 12x) and the superficial cervical lymph nodes (scLNs) (zoom 8x) were acquired (with an exposure time of 500 ms) as well as of the saphenous vein (with an exposure time of 1000 ms, zoom 40x, respectively). Tracer signal intensity of these locations served as a quality control for the tracer infusion, as well as a means of acquiring additional information on CSF flow through the central canal, efflux to scLNs, and amount returned to the blood circulation. Lack of P40D800 tracer signals in scLNs was regarded as failed infusion, therefore data from these mice were discarded. The autofluorescence signal of the skin on the green channel was used to precisely locate the mouse under the microscope.

### Assessment of tracer signals at the thoracic spine, sacral spine, saphenous vein and scLNs

A circular region of interest of radius 2.5 mm was placed over the thoracic region on the acquired videos. Using the “measure profile” function from ZEN 2.6 software, a table of fluorescence intensity in counts vs. time in minutes (min) was exported into Microsoft Excel. To quantify the fluorescent signal enhancement at 60 min post infusion, the average value from three control mice without tracer infusion were used as baseline level. This baseline intensity was then subtracted from the fluorescence intensity values acquired at 60 min post infusion. Same procedures were used to assess tracer transport to sacral spine, saphenous vein and scLNs, except that the circular region of interest of radius was 1.2 mm, 0.15 mm and 0.8 mm, respectively.

### Tissue processing

After intravital NIR imaging, all mice were subjected to transcardial perfusion with 10 ml PBS (Gibco, Paisley, UK) followed by 10 ml of 4% paraformaldehyde (PFA, Merk Darmstadt, Germany) in PBS. Cranium and spine tissues were harvested and post-fixed in 4% PFA for 24 h, then decalcified in 14% ethylenediaminetetraacetic acid (EDTA, Sigma-Aldrich, St. Louis, MO, USA) in PBS for 7–10 days. All tissues were immersed in sucrose (Merk, Darmstadt, Germany) 30% in PBS for 3 days for cryoprotection before being frozen at − 80 °C in O.C.T. (Tissue-Tek^®^, Sakura Finetek, Umkirch, Germany). Spines were sectioned by a surgical blade into four segments (cervical, thoracic, lumbar and sacral) using vertebral bodies T1, T13 and L4 as landmarks. The cervical segment contains the vertebral column above T1, the thoracic segment contains the vertebral column between T1 and T13, the lumbar segment contains the vertebral column between L1 and L4, and the sacral segment contains the caudal end of vertebral column below L4. 30-µm thick sections were cut using a cryostat (CryoStar, NX50, Epredia, Cham, Switzerland) and were stored at -20 °C for later immunofluorescence staining.

### Immunofluorescence staining and confocal imaging

All the immunofluorescence stainings were performed on tissues from EAE mice that were not subjected to the i.c.v-infusion of OVA-AF647 or liposomes. Frozen tissue sections were first hydrated with PBS for 10 min, then permeabilized by 0.1% triton for 10 min. 10% goat or donkey serum (depending on the species where secondary antibodies were raised) was used for blocking for 1 h at room temperature. Sections were incubated with primary antibodies with appropriate dilutions for 3 h at room temperature and then washed with PBS before incubating with appropriate secondary antibodies for 2 h at room temperature. Primary antibodies used in this study were: Fibrinogen (Rabbit polyclonal IgG, 1:200 dilution, LSBio), GFAP (Rabbit polyclonal IgG, 1:200 dilution, Dako), Laminin (Rabbit polyclonal to Laminin 1 + 2, 1:100 dilution, Abcam), E-cadherin (Goat polyclonal IgG, 1:100 dilution, R&D systems). LYVE-1 (rabbit IgG, AngioBio, catalog 11–034, 1:600 dilution). Secondary antibodies used in this study were: Donkey anti-rabbit IgG Cy5 (1:300 dilution, Jackson ImmunoResearch Laboratories, Inc.), Donkey anti-goat IgG Alexo Fluor 647 (1:500 dilution, Jackson ImmunoResearch Laboratories, Inc.). Overview of spinal cord and brain sections were imaged under Zeiss Axiozoom V16 microscope. Higher magnification images were acquired under LSM800 Zeiss confocal microscope.

### Quantification of OVA-AF647 signal within SAS and central canal

To quantify the OVA-AF647 mean fluorescence intensity, all images analyzed were taken with the same settings under the Zeiss AxioZoom.V16 microscope. Four rectangular ROIs of 200 × 20 μm were measured from the dorsal, left lateral, right lateral, and ventral aspects of the surface of the spinal cord in decalcified tissue. The average of the four measured values accounts for one data point from one tissue section. To quantify the OVA-AF647 mean fluorescence intensity, one circular ROI of 50 μm diameter placed on the central canal was measured. For the statistical analyses of each segment of spinal cord, 10–15 tissue sections containing that specific segment were randomly chosen from each mouse, and 3 mice were included in each experimental group.

### Quantification of cell infiltrates and the size of sacral central canal

Quantification of CCR2^+^ cell infiltrates in relation to the area of the spinal cord on cervical, thoracic, lumbar and sacral sections was assessed in semi-automated fashion with Fiji [83]. In brief, channels were split, and the red channel converted to a 16-bit grayscale image. After filtering for optimizing automatic thresholding and masking functions the spinal cord was circled and the CCR2^+^ area as a percentage of the whole spinal cord was calculated according to predefined threshold values set (by L.X. and A.M.) 30–60 randomly chosen sections of each spinal cord segment (cervical, thoracic, lumbar and sacral) from 3 to 6 animals per experimental group were analyzed. The same set of images from the sacral spinal cord used for quantifying the CCR2^+^ cell infiltrates were also used to measure the size of central canal. The area of central canal was measured in ZEN3.2 software by manually contouring the wall of central canal based on DAPI signals.

### Brain water content measurements

Naïve mice and aEAE mice were euthanized with CO_2_ followed by brain and spinal cord harvest. The spinal cord was further divided into rostral and caudal halves. Tissue was weighed (Mettler AE50, Greifensee, Switzerland) and transferred to an oven for 48 h at 90 °C for drying and subsequent reweighing. Water content was calculated as (weight at 48 h-initial weight)/initial weight.

### Quantification of LYVE-1^+^ lymphatic vessel area

30-µm thick coronal decalcified sections of the olfactory bulb region were collected. From the first section where olfactory bulb was visible until the first section that showed a lack of olfactory nerve fibers passing through cribriform plate, around 50 tissue sections were collected. 24 tissue sections spanning the rostral, middle and caudal olfactory bulb regions were selected from each experimental group (3 animals/group) and LYVE-1^+^ immunofluorescent staining was performed. Calculation of LYVE-1^+^ area was done in a semi-automated in Fiji using a script written by A.M. The area of interest for above (including those passing through) and below cribriform plate was manually drawn based on the DAPI signal.

### Statistics

Statistical analyses were performed with GraphPad Prism 9 (La Jolla, CA, USA), Graphs and values in the texts represent mean ± SD. Outliers were detected by ROUT method and removed from further analysis. All groups were found to be normally distributed using the Kolmogorov–Smirnov (K–S) test. Means of two groups were compared with the two-tailed Student’s t test. Means of multiple groups were compared with the one-way ANOVA with the Dunnett’s or Turkey multiple comparison post hoc test. A *p*-value < 0.05 was considered statistically significant.

## Electronic supplementary material

Below is the link to the electronic supplementary material.

Supplementary Material 1

Supplementary Material 2

Supplementary Material 3

Supplementary Material 4

## Data Availability

All underlying data of this study will be made available from the corresponding author upon reasonable request.

## Abbreviations

SAS: Subarachnoid space
EAE: Experimental autoimmune encephalomyelitis
CSF: Cerebrospinal fluid
NIR: Near-infrared
MS: Multiple sclerosis
CNS: Central nervous system
RRMS: relapse remitting MS
BBB: blood-brain barrier
CFA: Complete Freund’s adjuvant
PTX: Pertussis toxin
i.c.v: intracerebroventricular infusion
aEAE: Active experimental autoimmune encephalomyelitis
MOG_35 − 55_: Myelin oligodendrocyte glycoprotein peptide
OVA-AF647: Alexa647-conjugated ovalbumin
scLNs: superficial cervical lymph nodes
dcLNs: deep cervical lymph nodes
EDTA: Ethylenediaminetetraacetic acid
GFAP: Glial fibrillary acidic protein
LVs: Lymphatic vessels
CP: Cribriform plate
p.i.: post-immunization day
LYVE-1: lymphatic vessel endothelial hyaluronan receptor 1
ALS: amyotrophic lateral sclerosis
