# Supplementary material for: Impairment of spinal CSF flow precedes immune cell infiltration in an active EAE model
Source: J Neuroinflammation. 2024 Oct 23;21:272. doi: 10.1186/s12974-024-03247-9 (PMC11520187; doi:10.1186/s12974-024-03247-9)
Supplement: Supplementary file 4 — Supplementary Material 4 [file 12974_2024_3247_MOESM4_ESM.pdf]

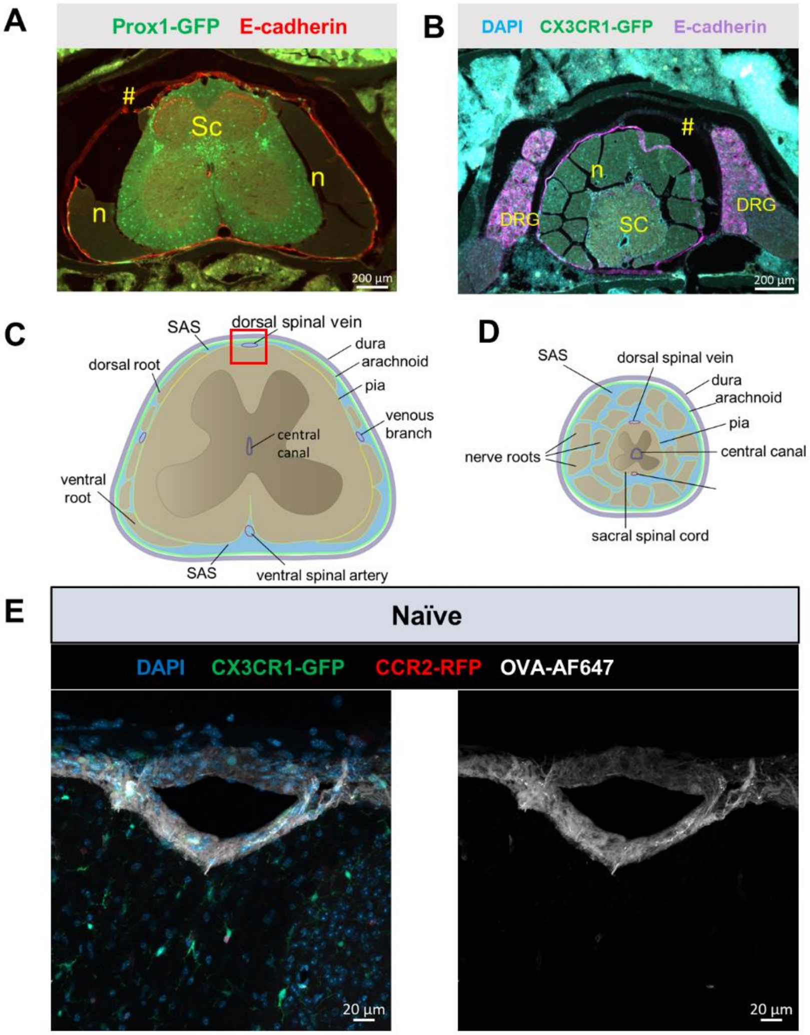

Supplemental Fig. 1 | E-cadherin staining confirms the external border of SAS. (A) Representative overview image of the decalcified lumbar spine from a Prox1-GFP mouse with E-cadherin staining marking the arachnoid barrier layer. E-cadherin layer is adjacent to the Prox1-EGFP signal, which covers the surface of spinal cord (SC) and the spinal nerve roots (n). #, dura mater. **(B)** Representative overview image of the decalcified sacral spine, showing the E-cadherin layer is below the dura mater (#) and covering the nerve bundles (n), which are surrounding the sacral spinal cord (note that E-cadherin+ layer falls off during tissue processing). Note that some neurons inside the dorsal root ganglion (DRG) are also positive for E-cadherin staining. **(C, D)** Schematics of the lumbar and sacral spinal cord showing essential anatomical structures. **(E)**, representative confocal images of OVA647 signal within the subarachnoid space around the dorsal spinal vein.

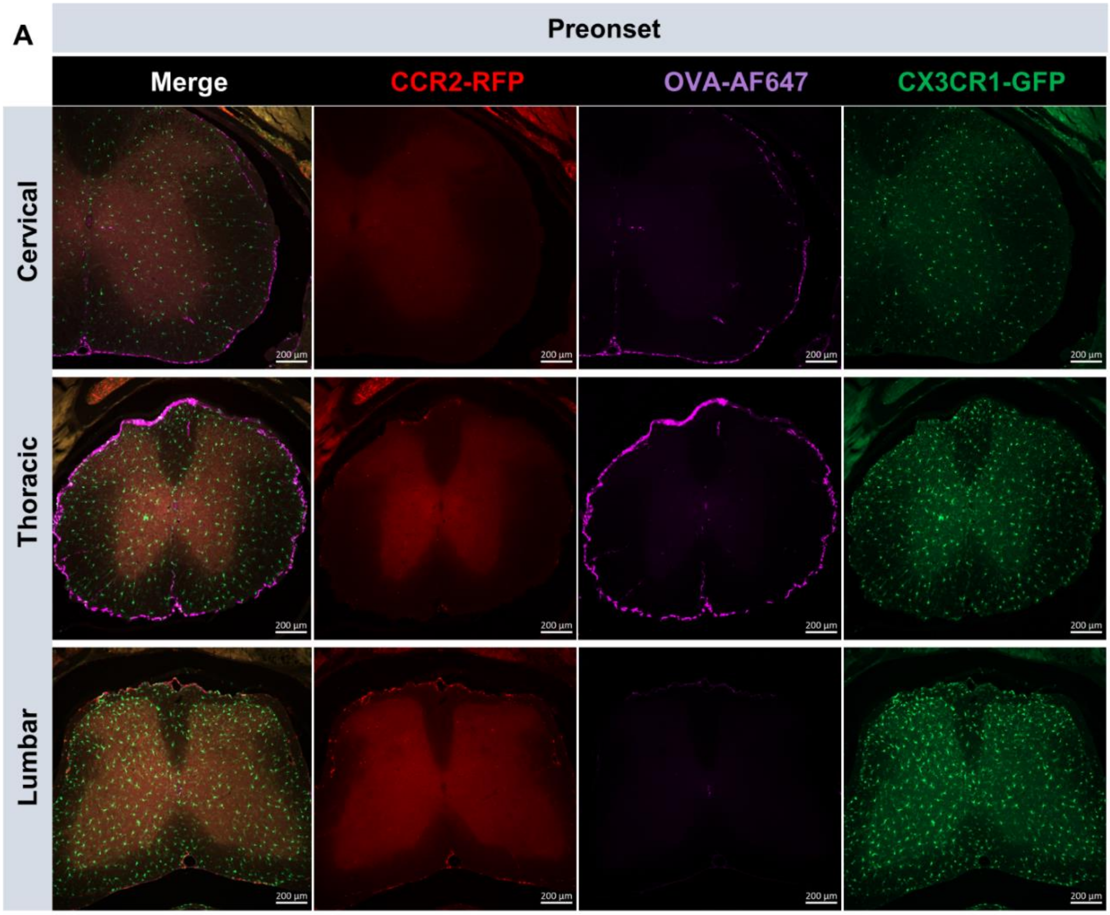

Supplemental Fig. 2 | No CCR2⁺ cell infiltration in the rostral spinal cord at preonset stage. (A) Representative overview images of the decalcified cervical, thoracic and lumbar spinal tissue showing minimal CCR2⁺ infiltrates on the surface of thoracic spinal cord, clearly visible on the lumbar spinal cord, but not on the surface of the cervical spinal cord segments.

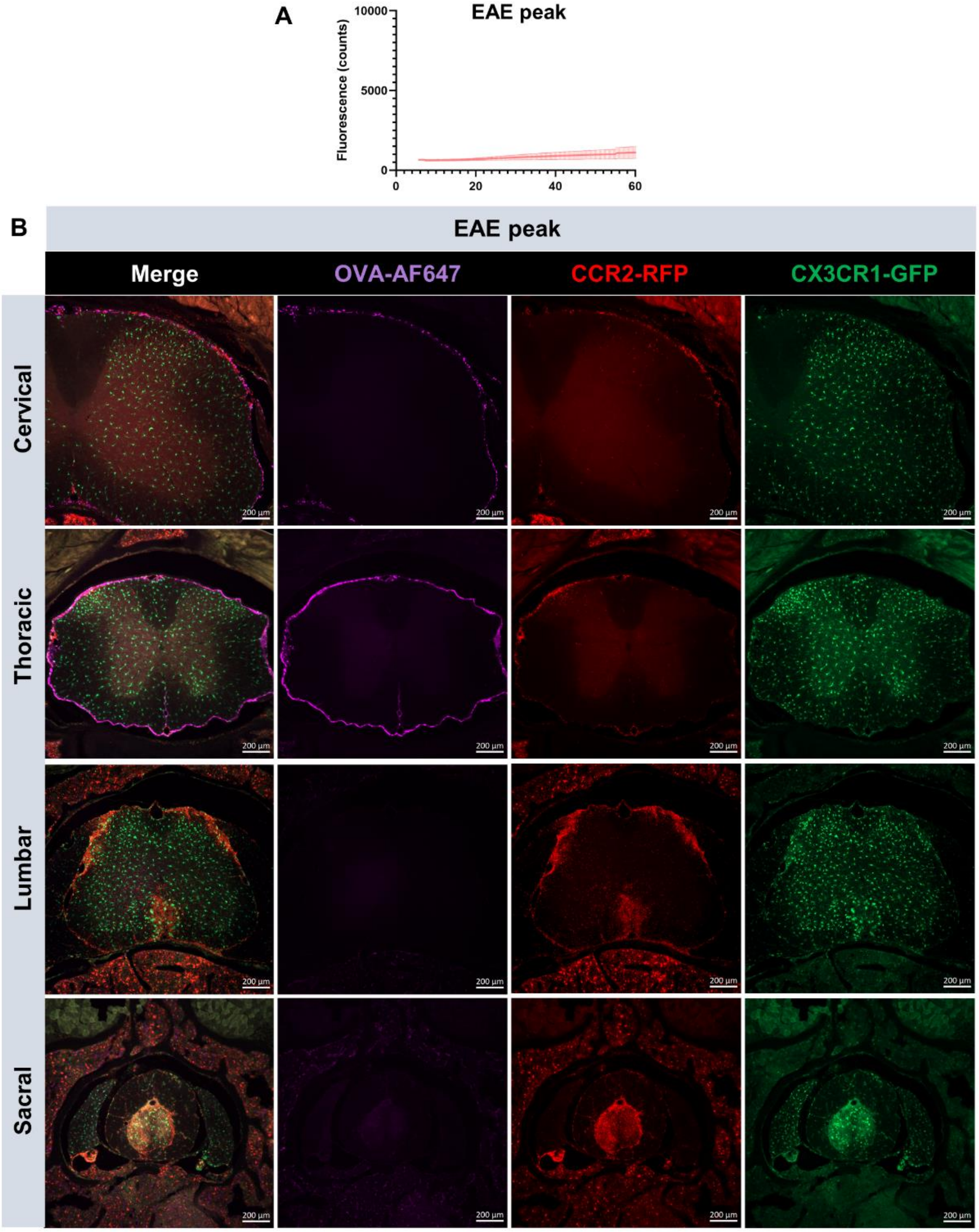

Supplemental Fig. 3 | CSF flow impairment and histopathological characteristics are independent of the immunization sites. (A) P40D800 tracer fluorescence signal intensity counts within the thoracic spine plotted against post-infusion time in shoulder control mice where MOG_{35-55}/CFA was injected subcutaneous near the shoulders. At EAE peak stage, all these mice showed impaired CSF flow (n=4). **(B)** Representative overview images of the decalcified cervical, thoracic and lumbar and sacral spinal tissue from one of the mice that data from panel A were acquired. Note that OVA-AF647 tracer signal is invisible at lumbar and sacral spinal cord. The nerve roots seem to merge at sacral spinal cord.

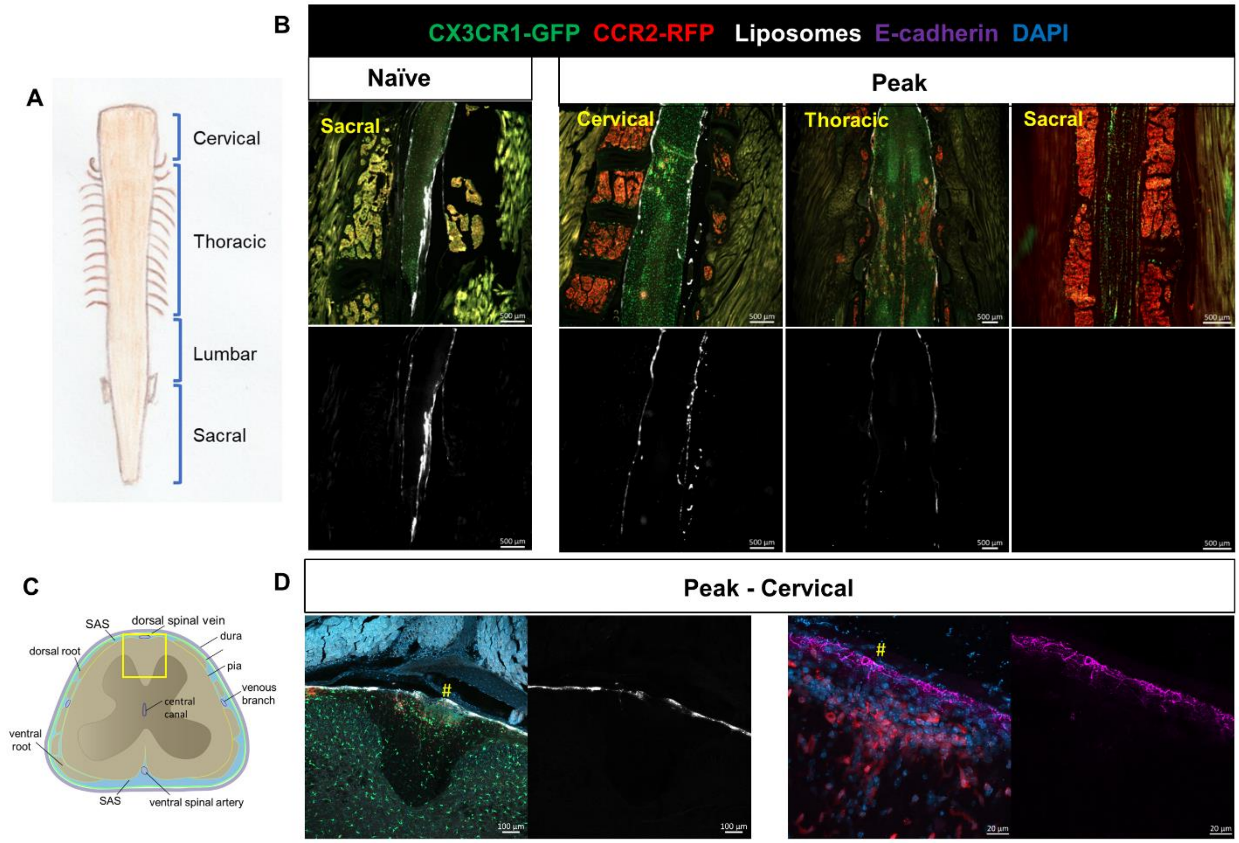

Supplemental Fig. 4 | Impairment of CSF flow along spinal cord at EAE peak confirmed by i.c.v-infused liposomes. (A) Schematic of the four segments of spinal cord. **(B)** Representative images of the distribution of liposomes in the spinal SAS from longitudinal sections of decalcified spinal columns. Liposomes are visible at the sacral spinal cord of a naïve mouse, but only found at cervical and thoracic segments of the spinal cord of a mouse at EAE peak stage. **(C)** Schematic of the lumbar spinal cord showing the region (yellow box) that the images from **(D)** were taken. **(D**) Left two panels, representative images of the dorsal aspect of a cervical spinal cord from a mouse at EAE peak stage showing liposomes do not spread into dura mater (#). Right panels, representative images of E-cadherin staining on the cervical spinal cord of a mouse at EAE peak stage showing CCR2^{+} infiltrates underneath the E-cadherin layer.

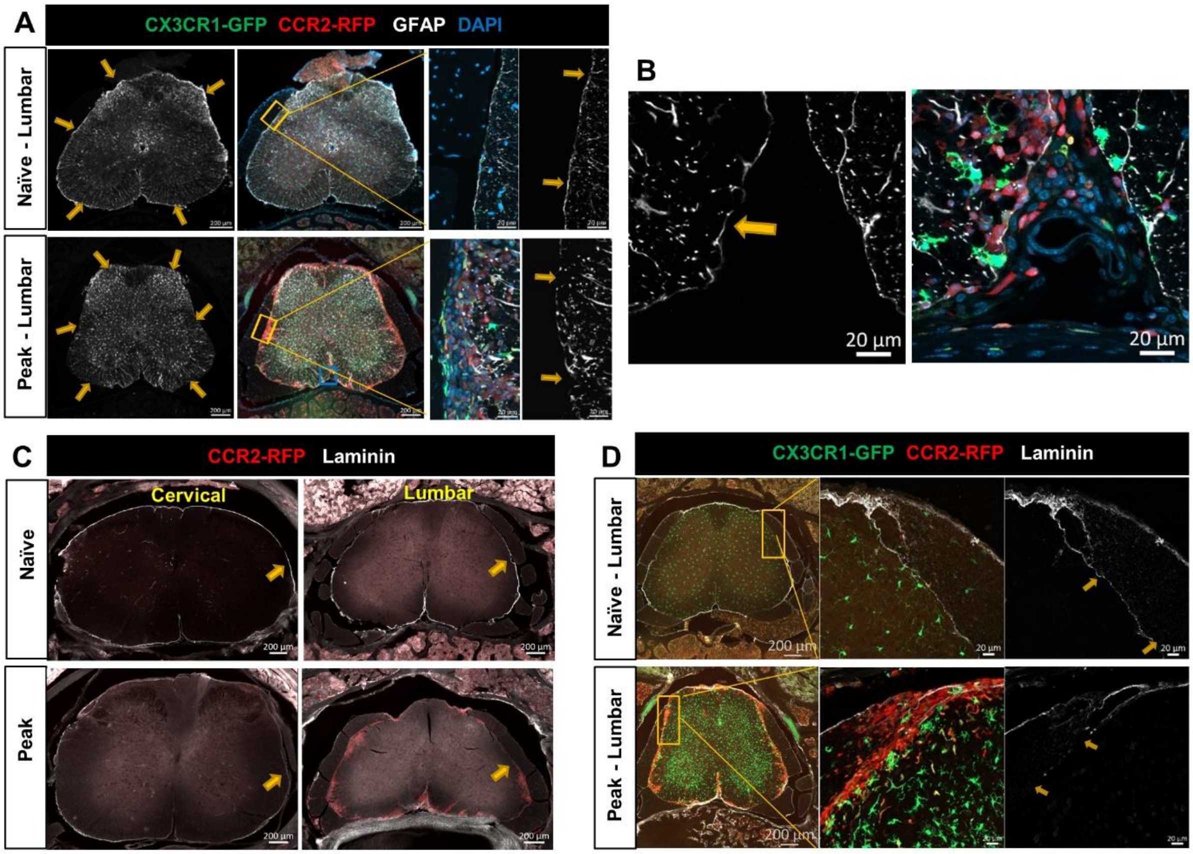

Supplemental Fig. 5 | GFAP and laminin staining confirms that the breakdown of glia limitans superficialis occurs at EAE peak phase. (**A)** Overview images of GFAP staining show a clear outline of the lumbar spinal cord of a naïve mouse, which is missing in the same region of a mouse with peak EAE (arrows). **(B)** Confocal images show the association between the discontinuous GFAP staining (arrow) and CCR2^{+} immune cell infiltrates (blue box in A, peak-lumbar). **(C)** Representative overview images of laminin staining on the cervical and lumber spinal cord segments of naïve and EAE peak mice. The surface of the spinal cord (arrows) shows positive laminin immunoreactivity except for that from the lumbar segment at EAE peak. **(D)** Confocal images of laminin staining (middle and right panels) of the boxed regions of the overview image (left panels) obtained from the lumbar spinal cords of mice (naïve and peak EAE). Note the continuous line of laminin between spinal cord surface and dorsal root in naïve animal, which is discontinuous in EAE peak animal.

Supplemental Video Legends

Supplemental video 1 | P40D400 tracer signal in thoracic spine increases with time in a naïve mice. There is a delay before P40D800 tracer signal continuously increases within the 60 min post-i.c.v infusion. The time indicated on the video is the actual acquisition time. The end of the video is 60 min post-i.c.v. infusion.

Supplemental video 2 | P40D800 tracer signal in thoracic spine does not increase with time in a mouse at EAE peak stage. There is no visible change in P40800 tracer signals within the 60 min post-i.c.v infusion. The time indicated on the video is the actual acquisition time. The end of the video is 60 min post-i.c.v. infusion.

Supplemental video 3 |Strong fibrin(ogen) immunoreactivity in the leptomeninges near the dorsal spinal vein. A mesh-like network of fibrin(ogen) is visible in the leptomeninges near the dorsal spinal vein of mouse at preonset stage. $CCR2^+$ cells are located in the gaps within the fibrin mesh.
